# Three-dimensional multiple object tracking (3D-MOT) performance in young soccer players: Age-related development and training effectiveness

**DOI:** 10.1371/journal.pone.0312051

**Published:** 2025-08-01

**Authors:** Yulu Zhang, Yu Zhang, Qi Zhang, Xingyu Pan, Gang Xu, Jie Li

**Affiliations:** 1 Sports Coaching College, Beijing Sport University, Beijing, China; 2 School of Psychology, Beijing Sport University, Beijing, China; 3 Center for Cognition and Brain Disorders, School of Clinical Medicine, Hangzhou Normal University, Hangzhou, China; Instituto Politecnico de Santarem Escola Superior de Desporto de Rio Maior, PORTUGAL

## Abstract

**Objectives:**

The aim of the present study is to examine the developmental trajectory of 3D-MOT performance in young soccer players, and to investigate the age-related 3D-MOT performance training effect.

**Methods:**

First, Experiment 1 assessed 3D-MOT performance of 404 male soccer players aged 12–18 years. Then, 127 athletes in Experiment 1 aged 13, 15, and 17 were selected as participants in Experiment 2, randomly assigned to intervention group and the control group. The intervention group but not the control group received 15 sessions of 3D-MOT training (3 sessions per day for 20 minutes for 5 days). Subsequently, post-test of 3D-MOT performance was conducted on the two groups.

**Results:**

Experiment 1 showed that there was a significant main effect of age on the peak speed (*p* < .001,η² = .041) of 3D-MOT performance, with the performance of participants aged 15 significantly higher than that of those aged 13 (*p* = .018, d = −0.559). Experiment 2 revealed a significant main effect of the intervention, including peak speed (*p* < .001), average speed(*p* < .001),and speed threshold(*p* < .001), but the interaction between the intervention and the age group was not significant.

**Conclusions:**

This research provide evidence that the 3D-MOT performance of young soccer players exhibits an increase between 13 and 15. Moreover, the results showed a significant training improvement in youth soccer players, but there was no significant relationship between the age-related development of 3D-MOT and the training effect in young soccer players. The above results provide new insights into soccer science and practice. Given the lack of data for those players younger than 12, this restricts the inference of whether there are potential sensitive periods. The study suggest that in the future, more evidence on athletes under the age of 12 be added to enhance the understanding of the full developmental trajectory of 3D-MOT performance in soccer players.

## Introduction

Due to the highly complex and dynamic nature of soccer playing environment, players must constantly engage in dynamic allocation of their visual attention to multiple regions of the field to gather necessary perceptual information to make effective responses [1–4]. For example, during a game, players have to simultaneously focus on multiple dynamic information within a short period of time, including the trajectory of the ball, the positions of their opponents, and the movements of their teammates.This information is crucial for players to make reasonable decisions. Therefore, the dynamic visual attention of soccer athletes has been the subject of significant research focus [5].

3D-MOT technology is an extension of the traditional MOT paradigm, aiming to simulate more realistic and complex motion scenarios [6]. The traditional paradigm of Multiple Object Tracking (MOT) mainly focuses on the two-dimensional plane [7], while 3D-MOT technology introduces depth information, making the tracking task closer to the motion situations in real life [8]. In a soccer match, players have to quickly and accurately judge the positions and movement trajectories of the ball, teammates, and opponents in the three-dimensional space. Therefore, 3D-MOT technology provides an effective tool for evaluating the dynamic visual attention of soccer players in complex visual environments [9]. The effectiveness of 3D-MOT assessment technology in sports has been extensively investigated. In a seminal study of athletes, Faubert utilized the 3D-MOT task to illustrate that professional team athletes (including those in soccer, rugby, and ice hockey) exhibited superior tracking abilities compared to athletes at the semi-professional level and non-athlete university students[9]. Moreover, Mangine et al. further demonstrated that the performance of the 3D-MOT task was strongly associated with the visual processing of players and the ability to respond to diverse stimuli on the basketball court [10]. Fleddermann et al reported positive results for the near-transfer effects of 3D-MOT training on processing speed and sustained attention in volleyball players [11]. Similar results were also observed in youth football players. Ehmann et al found that elite young players performed significantly better than sub-elite players in a 360°-MOT task [12].

Moreover, the relationship between growth, maturity status, and young players’ functional capacities has been an important concern to both researchers and coaches in the realm of sports. For example, Balyi and Hamilton proposed the “Long-Term Athlete Development” (LTAD) training model based on physiological principles [13]. Studies on LTAD have highlighted the presence of a sensitive period for general motor abilities, the non-linear development of multiple subsystems, and an accelerated improvement in measures of strength, speed, and endurance during these “windows of opportunity” [14,15,16]. Finding from these studies suggest that there are certain critical periods in the development of athletes in which they may experience rapid gains in physical abilities. During childhood and adolescence, targeted ability training can be particularly effective for improving physical characteristics [14,17,18]. On the other hand, previous research on adolescent cognitive development has shown across human development, adolescence is a period characterized by transformations towards life independence [19], and is a crucial phase for the development of many cognitive abilities [13]. Previous studies have suggested that the results of cognitive function tests can predict the success of top soccer players and that higher-order cognitive functions are believed to be related to talent identification and development in young soccer players [20]. For instance, Ehmann et al. revealed that there was a significant positive correlation between the performance of the 360°environmental MOT task and the accuracy score in the 360°soccer passing task, as well as the defensive performance score in the small-field game [21]. Moreover, elite youth players demonstrated better tracking performance than sub-elite youth players [12]. During this period, it can thus be speculated that the development of dynamic visual attention might influence sport performance and their potential for future enhancement. In light of the importance of cognitive capabilities in sports [22], especially the function of dynamic visual attention in soccer, it is of paramount importance to comprehend the development of these processes in young players. Based on the above content, a question of this study is triggered: whether athletes’ perceptual cognitive abilities have a similar “window period” for development as their physical abilities. It is worth noting that the majority of studies have only evaluated the impact of age on either MOT performance or training outcomes, with little attention paid to the interplay between these factors [12,21,23].

To summarize, considering the aforementioned research findings and limitations [24,25,19], this study aimed to (i) examine the age-related development of 3D-MOT performance in youth soccer players; and (ii) explore whether there are differences in the effect of 3D-MOT training among youth soccer players of different ages. In this way this research hoped to explore, for the first time, the question of how age interacts with perceptual-cognitive training in soccer players. To solve the above questions, this study conducted two experiments. Experiment 1 examined the performance of 12–18-year-old soccer players in the 3D-MOT task (Experiment 1). In Experiment 2, from the participants of Experiment 1, this study selected those aged 13, 15, and 17 as participants in Experiment 2 to investigate training effects across these three age groups. This research anticipates that the performance of 3D-MOT will improve with age, but the growth plateaus after the age of 15.and the 3D-MOT training effect may be less pronounced after age 15 [22].

## Experiment 1

### Methods

#### Participants.

The experiment involved 404 male youth soccer players from a soccer youth training center in China, aged between 12 and 18 (M = 15.34, *SD* = 1.65), who were divided into seven age groups: 12-year-old (n = 16), 13-year-old (n = 55), 14 -year-old (n = 45), 15-year-old (n = 106), 16-year-old (n = 65), 17-year-old (n = 75), and 18-year-old (n = 42). All participants in this study were athletes who had trained at the center for 1–3 years (M = 2.27, *SD* = 0.75), depending on their age (i.e., ages 12–13 had 1 year, ages 14–15 had 2 years, and ages 16–18 had 3 years). The training regimen was standardized for all players, consisting of one hour of physical training (e.g., strength and speed work) and two hours of on-field training per day (e.g., ball passing and dribbling). All participants had normal or corrected-to-normal vision, and normal color vision, and were unaware of the experiment’s purpose.

This study has been approved by the Institutional Ethics Committee of local university. Before the commencement of the study, permission to invite athletes was obtained from the coaches of soccer teams. All participants and their legal guardians provided written informed consent after receiving detailed information about the study procedure.

### Design and procedure

This study adopted a cross-sectional design, and the 3D-MOT task started on December 1, 2020, and ended on December 20, 2020 (01/12/2020-20/12/2020). All participants were tested individually in a quiet laboratory, with each participant completing the experiment independently. Before the experiment, participants were allowed to practice three times. Each participant was then asked to complete 30 trials of the 3D-MOT task, which was estimated to take 15 minutes per person. All participants followed the same standardized procedure and standing position to complete the trials. The first author of the paper was responsible for administering the tests according to the standardized instructions provided to the participants.

### Apparatus and stimuli

The 3D-MOT task was conducted using a fully immersive VR head-mounted display device (HTC VIVE) to create a three-dimensional VR scene. The HMD had a display resolution of 1,440 × 1,600 pixels per eye (2,880 × 1,600 pixels combined), a total field of view of 110°, and a refreshing rate of 90 Hz. The distance between the pupils could be adjusted for each participant. The stimulation area was a 5 × 5 × 2.5m cube room with a white wall and blue floor. The distance from the head-mounted display to the wall of the cube room was 4m, and the cross-section of the room measured 5 × 2.5m with a horizontal viewing angle of 43.6°and a vertical viewing angle of 22.6°. The stimuli consisted of 10 yellow 3D spheres with a diameter of 27.94 cm [26].

Throughout the experiment, participants utilized the VIVE control handle, which included the trigger button, touchpad button, and grip button, as reaction buttons for initiating, selecting, and confirming targets. The program control and data recording of the experiment were conducted using Steam VR 1.2.3 software.The technique has been used to prove effective in measuring dynamic attention in soccer players [26].

#### 3D-MOT task.

**Fig 1:**
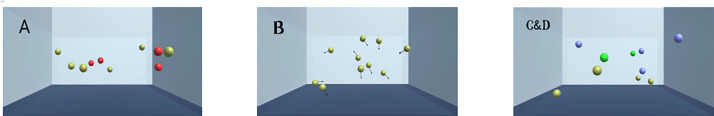
The three-dimensional multiple-object tracking (3D-MOT) task. The four stages of the 3D-MOT task: A is the target designating stage; B is the movement stage; C&D is the reaction and feedback stage.

The experimental procedure for the 3D-MOT task involved four stages (see Fig 1). The first stage was the target designating stage, which presented 10 spheres in the three-dimensional space and four spheres change to yellow as targets. The second stage was the movement stage, during which the target cue disappeared, the spheres designated as the targets returned to their original color, and all spheres moved randomly for 8 seconds. The third stage of the task involved a reaction stage in which all spheres stopped, and participants were instructed to identify the spheres designated as the targets in the first stage using a handle. In the fourth stage, which is the feedback stage, the designated target spheres were displayed in purple, while participants’ incorrect selections were displayed in green [16]. Furthermore, the experiment employed an adaptive procedure, consisting of 30 trials using a 1-up 1-down adaptive procedure.This method estimates the X•0 by calculating the midpoint of each pair of running peak and valley values, which is referred to as the midpoint running estimate. This approach is simple, robust, efficient and has a low estimation bias [27].The objects began with an initial movement speed of 0.68 m/s, which corresponded to 5.72°/s on the central section of the cubic space. If participants responded correctly on a trial, the objects’ movement speed was increased by 30% on the subsequent trial. Conversely, if participants responded incorrectly for any of the targets, the objects’ movement speed was reduced by 20%. The adaptive 3D-MOT task adopts the tracking speed of the targets as the dependent variable. Compared with the traditional paradigm that uses a constant velocity and takes the percentage of tracking accuracy as the dependent variable, the tracking speed can observe changes on a continuous proportional scale, thereby reflecting the performance differences of different groups in the tracking task more sensitively.Previous studies have shown that this adaptive adjustment can effectively measure the tracking speed of 3D-MOT tasks [8].

Currently, there are three speed measurements used for MOT tasks with an adaptive procedure, peak speed, average speed, speed threshold. The peak speed is the fastest speed at which a participant can correctly track all the targets, representing the maximum tracking speed achieved by participants during the 3D-MOT task [10]. The average speed means the overall mean of the speed across all 30 trials [28]. The speed threshold refers to the assumption that, after multiple responses to reversals in the task, the reversal gradually tends to stabilize. Therefore, the mean of the last four reversals when the reversal stabilizes in the later stages of the task is calculated as the speed threshold to reflect an individual’s tracking ability [8].

Whereas prior researchers have employed different speed measurements for MOT tasks, their sensitivity has not been examined. Therefore, this study analyzed all the three measurements.

### Data analysis

To assess the normality of data distributions, this study used the Shapiro–Wilk test. Although some age groups did not conform to the normal distribution, the P-P plot indicated only a small deviation from a normal distribution. Thus, this study chose to use a one-way ANOVA, where age was the between-group variable and the dependent variable was 3D-MOT performance measurements, including peak speed, average speed, and speed threshold. Data were weighted by inverse sample sizes to balance age group contributions [29]. Effect sizes were reported in terms of partial eta squared (η²). We interpreted the magnitude of the effect size as small (η² ≤ 0.01), medium (η² ≤ 0.06), and large (η² ≤ 0.14),and for Cohen’s d are small (d ≤ 0.20); medium (d ≤ 0.50) and large (d ≤ 0.80) [30].For post-hoc comparison, the Bonferroni correction was used. The overall alpha level was fixed at 0.05. Data were analyzed using Statistical Package for the Social Sciences (SPSS) version 27 (IBM corp. Released, 2020).

## Results

**Fig 2:**
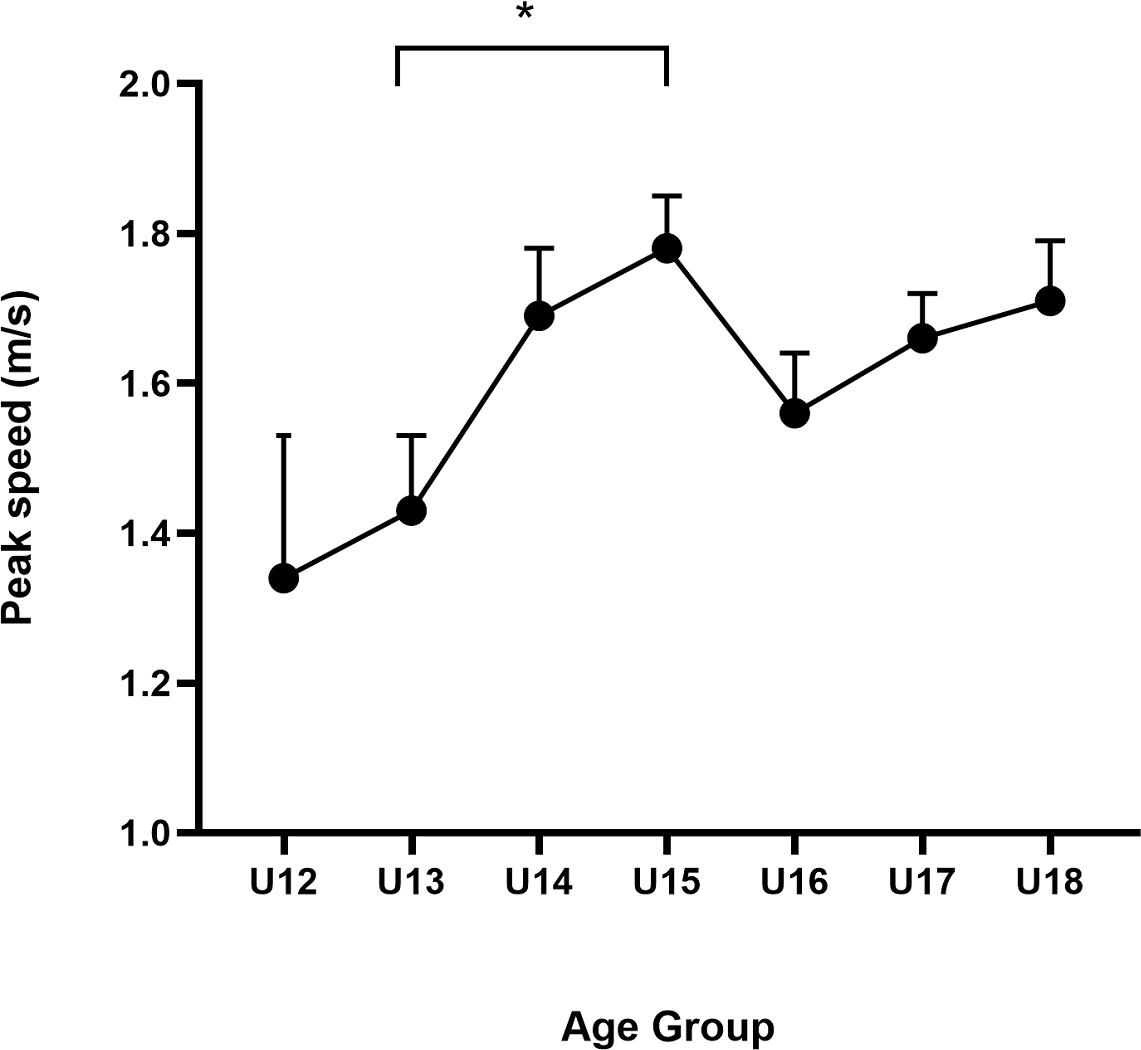
The peak speed of 3D-MOT for all age groups. ^*^p < .05.

The results for the three dependent variables in 3D-MOT for all groups are presented in Table 1. The ANOVA analysis revealed a significant main effect of age on peak speed, *F* (6,397) = 2.850, *p = *.010, 95%*CI* (0.003, 0.073), with a medium effect size (η² = .041) (see Fig 2), while no significant effects were observed in average speed, *F* (6,397) = 1.965, **p* *= .070, η² = .029, 95%*CI* (0.000, 0.055) and speed threshold *F* (6,397) =1.390, *p* = .217, η² = .021, 95%*CI* (0.000, 0.041). To correct the imbalance in sample sizes among the groups, a threefold weighting was applied to the 12-year-old group.The results after weighted cases showed that age had a significant main effect on peak speed, *F* (6,429) = 3.829, *p < *.001, 95%*CI* (0.009, 0.085), with a medium effect size (η² = .051), and average speed, *F* (6,429) = 2.480, **p* *= .023, η² = .034, 95%*CI* (0.000, 0.042),while no significant effects were observed in speed threshold *F* (6,429) =1.562, *p* = .157, η² = .021, 95%*CI* (0.002, 0.061).

**Table 1:** The descriptive statistics for all groups.

		Peak speed		Average speed		Speed threshold	
Group *(I)*	Group (*J)*	Mean difference (*I-J*) Std.Error	Sig. (d)& 95% CI	Mean difference (*I-J*) Std.Error	Sig. (d) & 95% CI	Mean difference (*I-J*) Std.Error	Sig. (d)& 95% CI
U12	U13	−0.088 (0.180)	1.000(−0.137) (−0.476,0.300)	−0.039 (0.134)	1.000(−0.083) (−0.448,0.370)	−0.033 (0.163)	1.000(−0.057) (−0.530,0.465)
	U14	−0.349 (0.184)	0.193(−0.543) (−0.756,0.059)	−0.212 (0.137)	1.000(−0.450) (−0.631,0.207)	−0.147 (0.167)	1.000(−0.257) (−0.657,0.363)
	U15	−0.442 (0.170)	0.002(−0.688) (−0.783,0.100)	−0.252 (0.126)	0.982(−0.535) (−0.638,0.134)	−0.229 (0.154)	1.000(−0.399) (−0.698,0.241)
	U16	−0.214 (0.177)	1.000(−0.333) (−0.588,0.159)	−0.123 (0.131)	1.000(−0.261) (−0.525,0.279)	−0.054 (0.160)	1.000(−0.094) (−0.543,0.435)
	U17	−0.313 (0.174)	0.182(−0.487) (−0.676,0.050)	−0.172 (0.130)	1.000(−0.365) (−0.568,0.225)	−0.106 (0.158)	1.000(−0.185) (−0.588,0.376)
	U18	−0.371 (0.186)	0.137(−0.578) (−0.786,0.044)	−0.251 (0.138)	1.000(−0.534) (−0.674,0.172)	−0.237 (0.168)	1.000(−0.415) (−0.752,0.277)
U13	U14	−0.261 (0.127)	0.929(−0.406) (−0.655,0.134)	−0.173 (0.095)	1.000(−0.367) (−0.462,0.117)	−0.114 (0.115)	1.000(−0.200) (−0.466,0.238)
	U15	−0.354 (0.105)^*^	0.021(−0.550) (−0.680,-0.027)	−0.213 (0.078)	0.143(−0.452) (−0.452,0.026)	−0.196 (0.095)	0.842(−0.342) (−0.487,0.095)
	U16	−0.126 (0.116)	1.000(−0.196) (−0.486,0.234)	−0.084 (0.086)	1.000(−0.178) (−0.348,0.180)	−0.021 (0.105)	1.000(−0.037) (−0.342,0.300)
	U17	−0.225 (0.112)	1.000(−0.350) (−0.573,0.124)	−0.133 (0.084)	1.000(−0.282) (−0.388,0.123)	−0.074 (0.102)	1.000(−0.128) (−0.384,0.237)
	U18	−0.283 (0.130)	0.677(−0.440) (−0.685,0.119)	−0.212 (0.096)	1.000(−0.451) (−0.507,0.083)	−0.205 (0.117)	1.000(−0.358) (−0.564,0.154)
U14	U15	−0.093 (0.113)	1.000(−0.145) −0.442,0.256)	−0.040 (0.084)	1.000(−0.085) (−0.296,0.216)	−0.082 (0.102)	1.000(−0.143) (−0.393,0.230)
	U16	0.135 (0.123)	1.000(−0.210) (−0.246,0.515)	0.089 (0.091)	1.000(0.189) (−0.190,0.368)	0.093 (0.111)	1.000(0.163) (−0.246,0.433)
	U17	0.036 (0.119)	1.000(−0.056) (−0.334,0.406)	0.04 (0.089)	1.000(0.085) (−0.231,0.312)	0.041 (0.108)	1.000(0.071) (−0.289,0.371)
	U18	−0.022 (0.136)	1.000(−0.035) (−0.444,0.399)	−0.039 (101)	1.000(−0.084) (−0.348,0.270)	−0.090 (0.123)	1.000(−0.158) (−0.466,0.285)
U15	U16	0.228 (0.1)	0.525(−0.354) (−0.082,0.537)	0.129 (0.074)	1.000(0.274) (−0.098,0.356)	0.175 (0.090)	1.000(0.305) (−0.101,0.451)
	U17	0.129 (0.095)	1.000(−0.201) (−0.167,0.425)	0.08 (0.071)	1.000(0.170) (−0.137,0.297)	0.123 (0.086)	1.000(0.214) (−0.142,0.387)
	U18	0.071 (0.115)	1.000(−0.110) (−0.287,0.429)	0.001 (0.086)	1.000(0.001) (−0.262,0.263)	−0.009 (0.104)	1.000(−0.015) (−0.328,0.311)
U16	U17	−0.099 (0.107)	1.000(−0.154) (−0.432,0.234)	−0.049 (0.080)	1.000(−0.103) (−0.293,0.195)	−0.052 (0.097)	1.000(−0.091) (−0.349,0.244)
	U18	−0.157 (0.125)	1.000(−0.244) (−0.546,0.232)	−0.128 (0.093)	1.000(−0.272) (−0.413,0.157)	−0.184 (0.113)	1.000(−0.321) (−0.530,0.163)
U17	U18	−0.058 (0.122)	1.000(−0.090) (−0.436,0.320)	−0.08 (0.091)	1.000(−0.169) (−0.457,0.198)	−0.131 (0.110)	1.000(−0.229) (−0.469,0.206)

Note: Peak speed = correctly identify the fastest speed of all four target balls with 100% accuracy;Average speed = the overall mean of these speed threshold measurements across all 60 trials; Speed threshold = the mean of the last four reversal in the task, *CI* = Confidence Intervals, F-values (^*^p < .05.), d = Cohen’s d

That means that the age group differences in peak speed are more sensitive than the differences in average speed and speed threshold.

Further pairwise analysis using Bonferroni’s post hoc test indicated that the peak speed of the 13-year-old group was significantly lower than that of the 15-year-old group (*p* = . 018, 95%*CI* [−0.675,-0.032]), with a medium effect size (d = −0.559). The weighted case results revealed that the 12-year-old group was significantly lower than the 15-year-old group (p = . 002, 95% CI [−0.7835, −0.100]), with a medium effect size (d = −0.668). Additionally, the 13-year-old group was significantly lower than the 15-year-old group (p = . 021, 95% CI [−0.680, −0.027]), with a medium effect size (d = −0.550). However, no significant differences were found between the 12 -year-old group and the 13-year-old group or between the age 14–18 group. That means the tracking performance had substantial improvement in the period from age 13–15.

The results indicate that there is some improvement in young soccer players’ 3D-MOT performance during adolescent, yet the improvement was not so dramatic, showing a relatively flat developmental trajectory. The peak tracking speed appears to be relatively sensitive to age changes, revealing a significant improvement from age 13–15.

## Experiment 2

The objective of Experiment 2 was to investigate the effectiveness of 3D-MOT training on young soccer players and examine whether there was an interaction between age and training effect.

In Experiment 1, significant differences in peak speed in 3D-MOT performance were observed in participants age 13 and 15. On the other hand, previous research has reported that the tracking performance of adolescent soccer players in 360°-MOT tasks exhibits a slowdown in growth after reaching the age of 16 [12]. Therefore, to investigate the relationship between 3D-MOT training and age-related development in greater depth, the participants of ages 13, 15, and 17 were selected for Experiment 2.

### Methods

#### Participants.

The sample size for this study was determined by estimating the effect size f using the G*power software 3.1.9.7 [31]. This study set the computation for a 2 × 3 analysis of variance (ANOVA) and set the expected effect size f at 0.25, theαlevel was set at 0.05, the desired power (1 − *β*) at 80%, and the correlation between repeated measures at 0.5. A total of 127 male youth soccer players from a certain youth soccer training center in China were recruited for this study (all players participated in Experiment 1).According to their age, participants were randomly assigned to either an intervention group or a control group, consisting of the 13-year-old intervention group (n = 20), the 13-year-old control group (n = 22), the 15-year-old intervention group (n = 20), the 15-year-old control group (n = 26), the 17-year-old intervention group (n = 20), and the 17-year-old control group (n = 19). Before the experiment, all participants had given their written informed consent to participate in the study. None of the subjects declared any problems with their eyesight, and they did not know the purpose of the experiment.

### Apparatus

Same as experiment 1.

### Procedure

#### 3D-MOT intervention group.

Short-term intervention has been proven to effectively improve 3D-MOT performance in previous studies. Therefore, the short-term training program in previous studies was followed in this study [9]. The specific plan is as follows: The intervention group received 15 sessions of active training for five consecutive days from December 23rd to December 28th, 2020 (24/12/2020–28/12/2020). They participated in three sessions of 3D-MOT task a day, of which, one session includes 30 trials.

#### Control group.

The control group participants did not receive any additional instruction or specific training beyond their regular training.

#### Pre-test and Post-test.

The pre-test results for Experiment 2 were obtained from the participants’ results in Experiment 1. After the five-day intervention period, all the participants completed one session of the 3D-MOT test the next day as the post-test.

### Data analysis

Data analysis was conducted using the 27th version of the Statistical Package for the Social Sciences (SPSS) (released by IBM Corporation in 2020). The 3D-MOT training performances were analyzed using a one-way ANOVA, where the age groups (13, 15, 17-year-old) was the between-group factor and the difference of 3D-MOT performance between the pre and post-test was the dependent variable. The training performance was assessed by calculating the difference between post-test values and pre-test values. To test the assumption of normality the Shapiro-Wilk test was performed, showing non-significant values for all the considered measures, thus confirming their normal distributions. Effect sizes were reported in terms of partial eta squared (η²).The effect size was categorized as small (η² ≤ 0.01), medium (η² ≤ 0.06), and large (η² ≤ 0.14), and for Cohen’s d are small ≤ 0.20; medium ≤ 0.50 and large ≤ 0.80 [29].For post-hoc comparison, the Bonferroni correction was used. The overall alpha level was fixed at 0.05.

### Results

**Fig 3:**
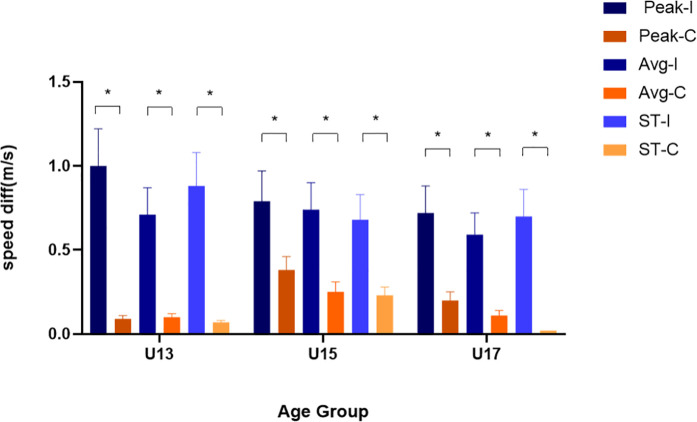
3D-MOT training effects of soccer players of 13, 15, and 17 years old. diff = the difference of post- and pre- tests; Peak-I = Peak speed of intervention group; Peak-C = Peak speed of control group; Avg-I = Average speed of intervention group; Avg-C = Average speed of control group; ST-I = speed threshold of intervention group, ST-C = speed threshold of control group; ^*^p **< .05.**

Fig 3 displays the results of 3D-MOT training effects for all groups, with the improvements between pre- and post-tests in the three speed measurements indicative of the training effects.

The results regarding peak speed showed a significant main effect of intervention, *F* (1,121) = 32.631, *p < *.001, 95%*CI* (0.092,0.327), with a large effect size (η² = 0.212). The improvement in peak speed achieved by the intervention group was found to be significantly higher than that of the control group (0.84 ± 0.08 vs. 0.22 ± 0.08, *p < *.001, 95%*CI* [0.643,1.396]), with a large effect size (*d* = 1.019). The result indicates that 3D-MOT training had a significant effect on improving the peak speed of young soccer players.The main effect of age was not significant, *F* (2,121) = 0.422, *p = *.657, η² = 0.005, 95%*CI* (0.000,0.004). The interaction effect between group and age was not significant, *F* (2,121)=1.989, *p = *.141,η² = 0.025, 95%*CI* (0.000,0.094).

The results revealed a significant main effect of intervention on average speed, *F* (1,121)=38.964, *p < *.001, 95%*CI*(0.153,0.399), with a large effect size (η² = .278). The improvement in average speed of the intervention group was significantly higher than that of the control group (0.68 ± 0.06 vs.0.16 ± 0.05, *p* *< *.001), with a large effect size (d = 1.234). The 3D-MOT training significantly improved the average speed of young soccer players.There was no significant age effect observed the interaction between age, *F* (1,121)=1.215, *p = *.300, η² = .014, 95%*CI* (0.000,0.070), and intervention was not significant, *F* (1,121)=0.325, *p = *.723, η² = .004, 95%*CI* (0.000,0.038).

The results regarding the speed threshold showed that there was a significant main effect of intervention, *F* (1,121) =38.941, *p < *.001, 95%*CI* (0.119,0.360), with a large effect size (η² = 0.239). The improvement in speed threshold of the intervention group was found to be significantly higher than that of the control group (0.75 ± 0.08 vs. 0.11 ± 0.07, *p < *.001, 95%*CI* [0.733,1.494]), with a large effect size (d = 1.113). The 3D-MOT training significantly improved the speed threshold of young soccer players. No significant age effect was observed and the interaction between age, *F* (1,121) =0.458, *p = *.634, η² = .006, 95%*CI* (0.000,0.046), and intervention was not significant, *F* (1,121) =1.127, *p = *.327, η² = .014, 95%*CI* (0.000,0.070).

In general, the results indicated that all groups improved in tracking performance after training. However, there was no differences in training effects among the three age groups.

## Discussion

The study assessed age-related development of 3D-MOT performance in soccer players aged 12–18 years old, as well as the relationship between age and 3D-MOT training in young soccer players. The results revealed a significant main effect of age in the peak speed of 3D-MOT performance but not the average speed or the speed threshold. Post-hoc tests found that the players aged 13 and 15 exhibited a significant difference in peak tracking speed, while none of the other age groups differed significantly. Regarding the 3D-MOT training effect, the results showed a significant improvement in 3D-MOT performance among youth soccer players following training. However, no significant interaction was detected between age and training.

Regarding the age-related development of 3D-MOT, the primary findings revealed that the peak speed of soccer players on the 3D-MOT task exhibited an age-related difference. A significant difference was evident between the players of age group 13 and those in age group 15. Furthermore, after weighting the 12-year-old group, the difference between it and the 15-year-old group reached a significant level; however, no significant differences were found between other age groups. These results suggest that the 3D-MOT performance of young soccer players improves to some extent during adolescence, but as a whole presents a relatively flat developmental trajectory from 12 to 18. The developmental evidence is consistent with earlier literature on development of other perceptual-cognitive skills in players [12,32]. For example, Beavan et al found that executive functions of young soccer players predominantly developed between 10 and 15 years of age, an observable plateau exists when players reach the age of 15, with only slight increases into early adulthood (18–21 years old) [21]. Previous studies investigating cognitive development in the general population have found that cognitive performance improves sharply in late childhood, then stabilizes during adolescence, and reaches adult levels after about age 14 [24,33,25]. The results showed a relatively flat developmental trajectory between the ages of 12 and 18, which may imply that the period of rapid growth in MOT ability in football players is before the age of 12. Given the lack of sample data of those under the age of 12 in this study, it has to some extent limited the inference of this possibility.Future research could explore the MOT development in younger children to provide a more comprehensive understanding of the age-related changes in MOT performance. Notably, no significant intergroup differences were observed between the age 12 group and other age groups. This might be attributed to limited statistical power resulting from the comparatively smaller sample size within the age 12 group (n = 16). Future studies could further expand the sample size, especially for the age group 12, to verify this hypothesis. This will help coaches to gain a profound insight of the developmental rules of MOT ability in youth soccer players and provide a fundamental reference for designing or developing training interventions to promote players to improve their visual attention ability in specific domains [34].

At present, there are mainly three computational methods for studying the tracking performance of 3D-MOT tasks. Firstly, the fastest speed among all the correct trials is taken as the peak speed representing the individual’s tracking ability [10]. Secondly, calculate the overall average of all trial reversals within the experiment as the average threshold to represent the individual’s tracking ability [28]. Finally, based on the assumption that an individual’s responses tend to stabilize in the later stage of the experiment, the average of the last four reversals of the task is taken (i.e., speed threshold) as the individual’s tracking ability [8]. This study found that the peak speed seems to be more sensitive to changes in players’ age than the average speed and the speed threshold. Peak speed represents the highest velocity that a player can track as the target object progressively accelerates. The sensitivity of peak speed to age-related changes may be attributed to its direct reflection of an individual’s maximum capacity and efficiency in processing visual information. As an indicator of an player’s ability to rapidly and accurately track multiple objects, peak speed highlights the cognitive and perceptual limits under high-demand conditions [10]. In contrast, average speed encompasses the speed of all object movements throughout the entire task, which may mask age-related differences that are more pronounced at the performance ceiling [28]. Similarly, the speed threshold, which is the average speed of the last four reversals of the object, may be influenced by factors unrelated to age, such as differences in individual task engagement, thereby attenuating age-related effects [9]. In sum, this provides novel evidence for future researchers to utilize the 3D-MOT task as an assessment tool.

Furthermore, the present study explored the relationship between age and training of 3D-MOT in young soccer players. Since a significant difference was found between the 13-year-old and the 15-year-old, and previous research observed that at the age of 16 soccer players exhibit a developmental plateau in their other perceptual-cognitive abilities [12,23], we selected a subset of players from the 13, 15, and 17-year-old groups in Experiment 1 as participants for Experiment 2. The main findings revealed a significant improvement in 3D-MOT performance, including the peak speed, the average speed, and the speed threshold. Previous research evidence has revealed the significant role of three-dimensional multi-object tracking (3D-MOT) in enhancing attention, visual information processing speed, and working memory [35,36].These findings suggest that regular training in 3D-MOT tasks may have a positive impact on the development of these cognitive skills in young soccer players. It is plausible that the improvements observed in Experiment 2 were due to the specific training regime that focused on enhancing MOT abilities [37]. This highlights the potential of targeted training interventions in facilitating the cognitive development of young players.

However, contrary to the expectations of this study, the situation that the training effect of 3D-MOT would weaken after the age of 15 has not been confirmed. One possible explanation is that the sensitive period for 3D-MOT training may extend beyond adolescence and cover adulthood and even old age [38]. This indicates that regardless of the age of the individual, 3D-MOT ability training for athletes can produce significant effects. Previous meta-analyses of the general population have shown that younger and older adults can achieve similar benefits when performing cognitive training [39]. Another possible explanation for the observed findings could be that the sensitive period for 3D-MOT training may occur during late childhood or preadolescence (8–12 years of age) [32,40].During this stage, the myelination process of the prefrontal-parietal network accelerates [41], and synaptic pruning leads to an improvement in neural efficiency [42], which may make the brain particularly sensitive to adaptive training in the allocation of attentional resources. Thus, initiating 3D-MOT training earlier in this sensitive period may yield more pronounced improvements in cognitive abilities. In view of the limitations of the research design of this study, future research can consider including younger participants, such as children aged 8–12, to explore whether there is a specific developmental “window period” for 3D-MOT training.

## Limitations and future directions

Currently, there is a lack of research on 3D-MOT training in youth soccer players. While this study investigated age trends and training performance in players aged 12–18, there are several limitations to our procedures that need to be addressed in future research. Firstly, our study only focused on the 3D-MOT performance of players aged 12–18 and did not include players below the age of 12. Additionally, the study intervention group only included players aged 13, 15, and 17, which may have contributed to the lack of expected results in terms of identifying “windows of opportunity” for the development of 3D-MOT ability. In the future, it may be beneficial to consider players who are younger than 12 years old. Secondlly, it should be noted that the subsample size of players U12 in the present study was relatively small from a statistical perspective. Therefore, caution is advised when interpreting some of the results obtained from these comparisons. Future research is recommended to recruit at least 33 participants to ensure a minimum statistical effect size, thereby enhancing the credibility of the research results. Thirdly, this study adopted a short-term intervention approach of MOT training for five consecutive days, three times a day, without considering the impact of cognitive training load on training outcomes. It is suggested that future research should explore the influence of cognitive training load to further optimize the training program [43].Finally, this study only explored the training effect of 3D-MOT on cognitive ability and did not involve the possible influence of factors such as emotion and motivation on the training effect. Future research can comprehensively consider these factors to more comprehensively evaluate the application effect of 3D-MOT training in young football players [44].

## Conclusion

To conclude, the results of the current study found significant differences in the peak tracking speed of 3D-MOT performance among soccer players aged 13 and 15 years, while no significance difference were observed between other age groups or when using the average speed and speed threshold as measurements. On the other hand, the results showed significant training improvement in 3D-MOT performance among youth soccer players at age 13, 15, and 17, but no significant interaction between age and training performance. Overall, these findings suggest that 3D-MOT performance in young soccer players aged 12–18 years shows improvement to some extent, despite a relatively flat developmental trajectory. Furthermore, the positive effects of 3D-MOT training remained consistent across the adolescent stage.These results provide valuable references for coaches, helping them understand the development of cognitive abilities of young soccer players and design diversified training plans that include cognitive stimulation to effectively enhance the cognitive abilities of players.

## Supporting information

S1 Data The young soccer players’ 3D-MOT task results. (XLSX)
