# Supplementary material for: Three-dimensional multiple object tracking (3D-MOT) performance in young soccer players: Age-related development and training effectiveness
Source: PLoS One. 2025 Aug 1;20(8):e0312051. doi: 10.1371/journal.pone.0312051 (PMC12316292; doi:10.1371/journal.pone.0312051)
Supplement: Attachment — Submitted filename: renamed_58796.docx [file pone.0312051.s003.docx]

#### Response to the Reviewer's Comments

Manuscript number：PONE-D-24-37901

Title:

Three-dimensional multiple object tracking (3D-MOT) performance in young soccer players: age-related development and training effectiveness

Dear Editors and Reviewers,

We appreciate the opportunity to revise our manuscript titled "Three-dimensional multiple object tracking (3D-MOT) performance in young soccer players: age-related development and training effectiveness". We are grateful for the insightful comments provided by the reviewers. Those comments are all valuable and very helpful for revising and improving our paper, as well as the important guiding significance to our researches. In the following, we have provided detailed responses to each of the reviewers' comments. Revised portion are marked in red in the paper. Additionally, we have conducted a comprehensive revision of the entire manuscript. In this response letter, the reviewers' comments are presented in italics, and our corresponding changes and additions to the manuscript are highlighted in red text. We have tried our best to make all the revisions clear, and we hope that the revised manuscript meets the requirements for publication.

Reviewer#1: **Major Weaknesses**

Comment 1: *Lack of Pre-12-Year-Old Data: The study focuses on players aged 12-18, but the absence of data from players younger than 12 limits the ability to identify potential sensitive periods for 3D-MOT development. This is a significant gap, as the authors suggest that rapid growth in MOT ability may occur before age 12 (Lines 306-311, Page 17).*

Response 1: First of all, we would like to express our sincere gratitude to the reviewer for your insightful comments and valuable feedback. The following is our response to this comment.

At the beginning of the research design, numerous literatures have indicated that the maturation process of the adolescent brain continues until the end of adolescence. For instance, the changes in the ratio of white matter to gray matter in the brain suggest that the brain structure still undergoes large-scale remodeling in late adolescence. In light of this, this study mainly delves into the age-related perceptual-cognitive ability changes of athletes from early adolescence to early adulthood. As the results did not meet our expectations, the hypothesis that the rapid growth of MOT ability may occur before the age of 12 was proposed in the discussion. Regarding this major limitation, we suggested in the research outlook section that future studies should focus on the cognitive ability development of athletes before the age of 12.

Reviewer#1: **Major Weaknesses**

Comment 2: *Small Sample Size for Younger Age Groups: The sample size for the 12-year-old group is notably small (n=16), which may affect the generalizability of the findings (Lines 91-94, Page 6).*

Response 2: We sincerely appreciate the valuable comment. The following is our response to this comment.

As the experiment was conducted during the COVID-19 pandemic, many players in the 12-year-old group were unable to participate, which to some extent limited the sample size. In response to this comment, we have included a discussion in our study on the impact of the relatively small sample size in the 12-year-old group on the research results.

The revised part now reads：“Notably, no significant intergroup differences were observed between the age 12 group and other age groups. This might be attributed to limited statistical power resulting from the comparatively smaller sample size within the age 12 group (n = 16). Future studies could further expand the sample size, especially for the age group 12, to verify this hypothesis. ” (Lines 339-343, Page 19,)

Reviewer#1: **Major Weaknesses**

Comment 3: *Limited Exploration of Transfer Effects: The study does not explore whether improvements in 3D-MOT performance transfer to actual soccer performance on the field. This limits the practical implications of the findings (Lines 329-352, Pages 19-20).*

Response 3: We sincerely appreciate the valuable comment. The following is our response to this comment.

The core objective of this study is to explore whether the improvement of players' cognitive abilities is associated with specific sensitive periods of age growth, rather than the transfer effect of training. In response to this comment, we have added in the discussion that future research should explore the transfer impact of 3D-MOT training on actual soccer performance.

Reviewer#1: **Major Weaknesses**

Comment 4:*Insufficient Discussion on Mechanisms: The paper lacks a detailed discussion on the underlying mechanisms that drive the observed improvements in 3D-MOT performance, particularly in relation to cognitive and neural development during adolescence.*

Response 4:We sincerely appreciate the valuable comment. The following is our response to this comment.

In response to this comment, we have added a discussion on the potential neural mechanisms underlying the improvement of 3D-MOT performance in our deliberations, and analyzed the connection between the cognitive and neural development processes during adolescence and the enhanced training effects of 3D-MOT.

The revised part now reads:“Previous research evidence has revealed the significant role of three-dimensional multi-object tracking (3D-MOT) in enhancing attention, visual information processing speed, and working memory[42,43].The brain imaging studies have further elucidated the neural mechanisms underlying the enhancement of cognitive abilities by 3D-MOT, particularly in the parietal and frontal lobes of the cerebral cortex, which are considered key regions for attention shifts and eye movement control[44].These brain pathways may play a role in football players' acquisition of game information during matches. In this sense, during the 3D-MOT process, the attentional tracking of multiple elements may overlap with the brain networks required to obtain key information during the player's competition. By training these brain networks, soccer players might be able to more effectively filter and track key information during the game, thereby making quick and accurate responses. In the future, it is necessary to combine image or electroencephalogram research to explore the transfer effect of 3D-MOT performance and the neural processes behind the improvement.( Lines 371-385, Page 21-22)

…… During this stage, the myelination process of the prefrontal-parietal network accelerates[46], and synaptic pruning leads to an improvement in neural efficiency[47], which may make the brain particularly sensitive to adaptive training in the allocation of attentional resources. Notably, the functional connectivity between the anterior cingulate cortex and the dorsolateral prefrontal cortex gradually matures during this period[48], facilitating more effective cognitive control and attention regulation. Thus, initiating 3D-MOT training earlier in this sensitive period may yield more pronounced improvements in cognitive abilities. ”( Lines 389-396, Page 22-23)

Reviewer#1: **Minor Weaknesses:**

Comment 1: *Repetition in Discussion: Some points in the discussion are repetitive, particularly regarding the flat developmental trajectory and the lack of interaction between age and training effects (Lines 287-289, Page 16; Lines 374-377, Page 21).*

Response 1: We sincerely appreciate the valuable comment. The following is our response to this comment.

We have deleted the repetitive content in the discussion.

Comment 2: *Clarification Needed on Speed Measurements: While the study uses peak speed, average speed, and speed threshold, the rationale for selecting these specific metrics and their sensitivity to age-related changes could be better explained (Lines 143-153, Page 9).*

Response 2: We sincerely appreciate the valuable comment. The following is our response to this comment.

We have added the missing content raised in the comments (the analysis of the basic principles of specific indicators and their sensitivity to age-related changes) in the discussion.

The revised part now reads：“It is noteworthy that the results of this study found that the peak tracking speed seems to be more sensitive to changes in athletes' age than the average speed and the speed threshold. The present study examined age-related changes in task performance using the three dependent variables and found that peak speed was more sensitive to age-related development than the other measurements. Peak speed represents the highest velocity that a player can track as the target object progressively accelerates. The sensitivity of peak speed to age-related changes may be attributed to its direct reflection of an individual's maximum capacity and efficiency in processing visual information. As an indicator of an player's ability to rapidly and accurately track multiple objects, peak speed highlights the cognitive and perceptual limits under high-demand conditions[10]. In contrast, average speed encompasses the speed of all object movements throughout the entire task, which may mask age-related differences that are more pronounced at the performance ceiling[37]. Similarly, the speed threshold, which is the average speed of the last four reversals of the object, may be influenced by factors unrelated to age, such as differences in individual task engagement, thereby attenuating age-related effects [17]. In sum, this provides novel evidence for future researchers to utilize the 3D-MOT task as an assessment tool. ”( Lines 347-363, Page 20-21)

Comment 3: *Cl Statistical Reporting: The statistical reporting could be more detailed, particularly in terms of effect sizes and confidence intervals, to provide a clearer understanding of the magnitude of the observed effects.*

Response 3: We sincerely appreciate the valuable comment. The following is our response to this comment.

In response to this comment, we have added confidence intervals in the results section. Given that we employed the analysis of variance method, the *ŋp^2^* index reflects the magnitude of the effect size.

The revised part now reads：

| Group | 3D-MOT performance | | | | | |
| --- | --- | --- | --- | --- | --- | --- |
| Speed | Peak *M*(*SD*) | 95% *CI* | Avg *M*(*SD*) | 95% *CI* | ST *M*(*SD*) | 95% *CI* |
| U12 | 1.34 (0.78) | 1.03,1.65 | 1.01 (0.65) | 0.78,1.25 | 1.22 (0.80) | 0.94,1.50 |
| U 13 | 1.43 (0.71)^a*^ | 1.26,1.60 | 1.05 (0.50) | 0.93,1.18 | 1.25 (0.66) | 1.10,1.41 |
| U 14 | 1.69 (0.60) | 1.51,1.88 | 1.23 (0.46) | 1.09,1.37 | 1.37 (0.55) | 1.20,1.54 |
| U 15 | 1.78 (0.67)^a*^ | 1.66,1.91 | 1.27 (0.49) | 1.18,1.36 | 1.45(0.58) | 1.34,1.56 |
| U 16 | 1.56 (0.61) | 1.40,1.71 | 1.14 (0.44) | 1.02,1.25 | 1.28 (0.54) | 1.14,1.42 |
| U 17 | 1.66 (0.56) | 1.51,1.80 | 1.19 (0.43) | 1.08,1.29 | 1.33 (0.52) | 1.20,1.46 |
| U 18 | 1.71 (0.52) | 1.52,1.91 | 1.27 (0.43) | 1.12,1.41 | 1.46 (0.47) | 1.29,1.63 |

“Further pairwise analysis using Bonferroni's post hoc test indicated that the peak speed of the 13-year-old group was significantly lower than that of the 15-year-old group (1.43vs 1.78, *p* =.018, 95%*CI* [-0.68,-0.32]).” (Lines 215[Table 1] and 225,Page 20-21)

Reviewer#1: **SPECIFIC COMMENTS**

Comment 1: *Abstract: The abstract provides a good summary of the study's objectives, methods, results, and conclusions. However, it could briefly mention the lack of data for players younger than 12 as a limitation (Lines 1-20, Page 2).*

Response 1:We sincerely appreciate the valuable comment. The following is our response to this comment.

The abstract section supplements the limitations of this study, namely, the data of players under the age of 12 were not included.

The revised part now reads：“We suggest that in the future, more evidence on athletes under the age of 12 be added to enhance the understanding of the full developmental trajectory of 3D-MOT performance in soccer players. ”(Lines 21-23, Page 3 )

Reviewer#1: **SPECIFIC COMMENTS**

Comment 2:*Introduction: Lines 22-26, Page 3: The introduction effectively sets the stage by highlighting the importance of dynamic visual attention in soccer. However, it could benefit from a more detailed discussion of how 3D-MOT specifically relates to soccer performance.*

Response 2:We sincerely appreciate the valuable comment. The following is our response to this comment.

Regarding this comment, we have added the relevant content in the first paragraph.

The revised part now reads: “For example, during a game, players have to simultaneously focus on multiple dynamic information within a short period of time, including the trajectory of the ball, the positions of their opponents, and the movements of their teammates.This information is crucial for players to make reasonable decisions. ”(Page 4, Lines 29-32)

Reviewer#1: **SPECIFIC COMMENTS**

Comment 3:*Introduction: Lines 40-56, Page 4: The discussion of 3D-MOT technology and its application in sports is well-presented. However, the authors could provide more context on how 3D-MOT differs from traditional MOT tasks in terms of cognitive demands.*

Response 3:We sincerely appreciate the valuable comment. The following is our response to this comment.

In the introduction section, we have added the differences between 3D-MOT and traditional MOT, as well as the application advantages of 3D-MOT.

The revised part now reads: “Although the above-mentioned studies have extensively validated the tracking performance of athletes using the MOT task, most of them still employ the traditional two-dimensional planar MOT task. In the real environment, three-dimensional spatial information serves as a crucial clue for the visual system to judge and estimate the movement of objects. Individuals' attentional processing of dynamic information often combines multiple depth cues such as monocular and binocular depth cues for judgment and estimation, and these depth cues can provide individuals with a strong sense of depth perception[15]. ”(Lines 49-56, Page 5)

Reviewer#1: **SPECIFIC COMMENTS**

Comment 4:*Introduction:Lines 60-75, Page 5: The introduction of the "Long-Term Athlete Development" (LTAD) model is relevant, but the connection between physical and cognitive development could be more explicitly linked to 3D-MOT performance.*

Response 4:We sincerely appreciate the valuable comment. The following is our response to this comment.

In response to this comment, we have added relevant discussions in the introduction.

The revised part now reads:“Previous studies have suggested that the results of cognitive function tests can predict the success of top soccer players and that higher-order cognitive functions are believed to be related to talent identification and development in young soccer players[28]. During this period, it can thus be speculated that the development of dynamic visual attention might influence sport performance and their potential for future enhancement. In light of the importance of cognitive capabilities in sports[29], especially the function of dynamic visual attention in soccer, it is of paramount importance to comprehend the development of these processes in young players. ”(Lines 89-97, Page 7)

Reviewer#1: **SPECIFIC COMMENTS**

Comment 5: *Methods: Lines 91-94, Page 6: The sample size for the 12-year-old group is small (n=16), which may limit the generalizability of the findings. The authors should address this limitation in the discussion.*

Response 5:We sincerely appreciate the valuable comment. The following is our response to this comment.

In response to this comment, we have included a discussion in our study on the impact of the relatively small sample size in the 12-year-old group on the research results.

The revised part now reads：“Notably, no significant intergroup differences were observed between the age 12 group and other age groups. This might be attributed to limited statistical power resulting from the comparatively smaller sample size within the age 12 group (n = 16). Future studies could further expand the sample size, especially for the age group 12, to verify this hypothesis” ( Lines 339-343, Page 20)

Reviewer#1: **SPECIFIC COMMENTS**

Comment 6: *Methods: Lines 108-122, Page 7: The description of the VR apparatus and stimuli is thorough, but the authors could provide more details on how the 3D-MOT task was validated for use with soccer players.*

Response 6:We sincerely appreciate the valuable comment. The following is our response to this comment.

With regard to this comment, we add relevant research content and references to confirm the effectiveness of the described technique in measuring dynamic visual attention ability of soccer players.

The revised part now reads: “The technique has been used to prove effective in measuring dynamic attention in soccer players[35] ”(Lines 147-148, Page 10)

Reviewer#1: **SPECIFIC COMMENTS**

Comment 7: *Methods: Lines 123-138, Page 8: The 3D-MOT task procedure is well-described, but the rationale for the adaptive procedure (1-up 1-down) could be better explained, particularly in relation to its sensitivity to age-related changes.*

Response 7:We sincerely appreciate the valuable comment. The following is our response to this comment.

In response to this comment, we have added relevant discussions on this content in the methods.

The revised part now reads: “The adaptive 3D-MOT task adopts the tracking speed of the targets as the dependent variable. Compared with the traditional paradigm that uses a constant velocity and takes the percentage of tracking accuracy as the dependent variable, the tracking speed can observe changes on a continuous proportional scale, thereby reflecting the performance differences of different groups in the tracking task more sensitively.Previous studies have shown that this adaptive adjustment can effectively measure the tracking speed of 3D-MOT tasks[16].”(Lines 164-171, Page 11)

Reviewer#1: **SPECIFIC COMMENTS**

Comment 8: *Results: Lines 177-189, Page 10: The results are clearly presented, but the authors should provide more detailed statistical reporting, including effect sizes and confidence intervals, to better convey the magnitude of the observed effects.*

Response 8:We sincerely appreciate the valuable comment. The following is our response to this comment.

In response to this comment, we have added confidence intervals in the results section. Given that we employed the analysis of variance method, the ŋ_p_^2^ index reflects the magnitude of the effect size.

This part of the response is consistent with the third comment raised by the reviewer regarding the minor weaknesses.(Lines 209 [Table 1] and 219, Page 20-21)

Reviewer#1: **SPECIFIC COMMENTS**

Comment 9: *Results: Lines 190-198, Page 12: The finding that peak speed is more sensitive to age-related changes than average speed or speed threshold is interesting, but the authors should discuss why this might be the case in more detail.*

Response 9:We sincerely appreciate the valuable comment. The following is our response to this comment.

In response to this comment, we have added an analysis and discussion in the discussion section on why peak speed is more sensitive to the age-related aspects of players compared to average speed or speed thresholds.

The revised part now reads: “It is noteworthy that the results of this study found that the peak tracking speed seems to be more sensitive to changes in athletes' age than the average speed and the speed threshold. The present study examined age-related changes in task performance using the three dependent variables and found that peak speed was more sensitive to age-related development than the other measurements. Peak speed represents the highest velocity that a player can track as the target object progressively accelerates. The sensitivity of peak speed to age-related changes may be attributed to its direct reflection of an individual's maximum capacity and efficiency in processing visual information. As an indicator of an player's ability to rapidly and accurately track multiple objects, peak speed highlights the cognitive and perceptual limits under high-demand conditions[10]. In contrast, average speed encompasses the speed of all object movements throughout the entire task, which may mask age-related differences that are more pronounced at the performance ceiling[34]. Similarly, the speed threshold, which is the average speed of the last four reversals of the object, may be influenced by factors unrelated to age, such as differences in individual task engagement,thereby attenuating age-related effects [16]. In sum, this provides novel evidence for future researchers to utilize the 3D-MOT task as an assessment tool. ”(Lines 347-363, Page 20-21 )

Reviewer#1: **SPECIFIC COMMENTS**

Comment 10: *Discussion: Lines 278-289, Page 16: The discussion of the age-related development of 3D-MOT performance is thorough, but the authors should address the potential implications of the flat developmental trajectory observed from ages 12 to 18.*

Response 10:We sincerely appreciate the valuable comment. The following is our response to this comment.

In response to this comment, this study has added a discussion in the discussion section on the potential impact of the observed phenomenon of a relatively flat development trajectory for players aged 12 to 18.

The revised part now reads:“This result indicates that the dynamic visual attention ability of adolescent soccer players may have experienced a significant development peak before the age of 12, followed by another improvement during adolescence, and then showed a stable development trend. Notably, no significant intergroup differences were observed between the age 12 group and other age groups. This might be attributed to limited statistical power resulting from the comparatively smaller sample size within the age 12 group (n = 16). Future studies could further expand the sample size, especially for the age group 12, to verify this hypothesis. This will help coaches to gain a profound insight of the developmental rules of MOT ability in youth soccer players and provide a fundamental reference for designing or developing training interventions to promote players to improve their visual attention ability in specific domains. ”(Lines 336-346, Page 20)

Reviewer#1: **SPECIFIC COMMENTS**

Comment 11: *Discussion: Lines 329-352, Pages 19-20: The discussion of training effects is well-argued, but the authors should explore the potential mechanisms underlying the observed improvements in 3D-MOT performance, particularly in relation to cognitive and neural development.*

Response 11:We sincerely appreciate the valuable comment. The following is our response to this comment.

In response to this comment, we have added relevant discussions on the content in the discussion section.

The revised part now reads: “Previous research evidence has revealed the significant role of three-dimensional multi-object tracking (3D-MOT) in enhancing attention, visual information processing speed, and working memory[42,43].The brain imaging studies have further elucidated the neural mechanisms underlying the enhancement of cognitive abilities by 3D-MOT, particularly in the parietal and frontal lobes of the cerebral cortex, which are considered key regions for attention shifts and eye movement control[44].These brain pathways may play a role in football players' acquisition of game information during matches. In this sense, during the 3D-MOT process, the attentional tracking of multiple elements may overlap with the brain networks required to obtain key information during the player's competition. By training these brain networks, soccer players might be able to more effectively filter and track key information during the game, thereby making quick and accurate responses. In the future, it is necessary to combine image or electroencephalogram research to explore the transfer effect of 3D-MOT performance and the neural processes behind the improvement.”(Lines 371-385, Page 21-22 )

Reviewer#1: **SPECIFIC COMMENTS**

Comment 12: *Discussion: Lines 354-366, Page 20: The limitations section is comprehensive, but the authors should also discuss the potential implications of the small sample size for the 12-year-old group.*

Response 12:We sincerely appreciate the valuable comment. The following is our response to this comment.

In response to this comment, we have added relevant discussions on the content in the limitations section.

The revised part now reads: “Additionally, it should be noted that the subsample size of players U12 in the present study was relatively small from a statistical perspective. Therefore, caution is advised when interpreting some of the results obtained from these comparisons. In the future, it is important to recruit larger subsamples to improve the validity and reliability of the findings. ” (Lines419-423, Page 24)

Reviewer#1: **SPECIFIC COMMENTS**

Comment 13: *Conclusion: Lines 367-377, Page 21: The conclusion effectively summarizes the key findings of the study, but it could briefly mention the need for future research to explore the transfer of 3D-MOT training to actual soccer performance.*

Response 13:We sincerely appreciate the valuable comment. The following is our response to this comment.

In the conclusion section, we added the content that future research is needed on the transfer of 3D-MOT training to actual soccer performance.

The revised part now reads: “Future research could further explore the transfer of 3D-MOT training into practical soccer performance.” (Lines432-433,Page 25 )

We tried our best to improve the manuscript and made some changes marked in red in revised paper which will not influence the content and framework of the paper. We appreciate for Editors and Reviewers' warm work earnestly, and hope the correction will meet with approval. Best regards!
