# Supplementary material for: Three-dimensional multiple object tracking (3D-MOT) performance in young soccer players: Age-related development and training effectiveness
Source: PLoS One. 2025 Aug 1;20(8):e0312051. doi: 10.1371/journal.pone.0312051 (PMC12316292; doi:10.1371/journal.pone.0312051)
Supplement: Attachment — Submitted filename: Response to Reviewers.docx [file pone.0312051.s004.docx]

#### Response to the Reviewer's Comments

Manuscript number：PONE-D-24-37901

Title:

Three-dimensional multiple object tracking (3D-MOT) performance in young soccer players: age-related development and training effectiveness

Dear Editors and Reviewers,

We express our gratitude to you for providing the chance to revise our manuscript titled "Three-dimensional multiple object tracking (3D-MOT) performance in young soccer players: age-related development and training effectiveness". We are grateful for the insightful comments provided by the reviewers. Those comments are all valuable and very helpful for revising and improving our paper, as well as the important guiding significance to our researches. In the following, we have provided detailed responses to each of the reviewers' comments. Revised portion are marked in red in the paper. Additionally, we have conducted a comprehensive revision of the entire manuscript. In this response letter, the reviewers' comments are presented in italics, and our corresponding changes and additions to the manuscript are highlighted in red text. We have tried our best to make all the revisions clear, and we hope that the revised manuscript meets the requirements for publication.

**Editor's comments：**

Comment 1: *I would recommend writing in the third person (to avoid expressions like "we aimed", "we suggest" etc., etc.);*

Response 1: First of all, we would like to express our sincere gratitude to the reviewer for your insightful comments and valuable feedback. The following is our response to this comment.

We will modify all the "We" in the article to the third person.

Comment 2: *abstract should present some statistics;.*

Response 2: We sincerely appreciate the valuable comment. The following is our response to this comment.

According to the reviewers' comment, we revised the abstract and added statistical data to the description of the results section.

The revised part now reads：“Results:Experiment 1 showed that there was a significant main effect of age on the peak speed (p <.001，η²=.041) of 3D-MOT performance, with the performance of participants aged 15 significantly higher than that of those aged 13 (p =.018，d=-0.559). Experiment 2 revealed a significant main effect of the intervention, including peak speed (p <.001), average speed(p <.001),, and speed threshold(p <.001), but the interaction between the intervention and the age group was not significant. ” (Lines 11-16, Page 2)

Comment 3: *please avoid using keywords already present in the title.*

Response 3: We sincerely appreciate the valuable comment. The following is our response to this comment.

According to the comment of the reviewers, we revised the key words.

The revised part now reads：“visual attention; soccer players;teenagers; cognitive training” (Lines 25, Page 2)

Comment 4: *please add a space when introducing citations in text (e.g., L28, the current form is "responses[1-4]", please amend to "responses [1-4]"). Please revise the full text;*

Response 4:We sincerely appreciate the valuable comment. The following is our response to this comment.

According to the reviewers' comment, we added spaces before the citations throughout the full text.

Comment 5: *Introduction is too long. Please reduce the text to two and half pages at maximum;.*

Response 5: We sincerely appreciate the valuable comment. The following is our response to this comment.

According to the comment of the reviewers, we moderately deleted the introduction part.

Comment 6: *before introducing "Experiment 1", it is suggested describing Study Design where it should also be explained that 2 experiments were conducted;.*

Response 6: We sincerely appreciate the valuable comment. The following is our response to this comment.

According to the reviewers' comment, we added the description of the experimental design in the introduction part.

The revised part now reads：“To solve the above questions, this study conducted two experiments. Experiment 1 examined the performance of 12 to 18-year-old soccer players in the 3D-MOT task (Experiment 1). In Experiment 2, from the participants of Experiment 1, this study selected those aged 13, 15, and 17 as participants in Experiment 2 to investigate training effects across these three age groups. ”( Lines 92-96, Page 5)

Comment 7: *In data analysis (for both experiments), please describe the thresholds used to interpret the effect sizes. In addition, effect sizes should be presented for Bonferroni comparisons;*

Response 7: We sincerely appreciate the valuable comment. The following is our response to this comment.

According to the comment of the reviewers, we have modified the results section.

The revised part now reads：

Experiment 1：The ANOVA analysis revealed a significant main effect of age on peak speed, F (6,397) = 2.850, p=.010, 95%CI (0.003, 0.073), with a medium effect size (η²=.041) (see Figure. 3), while no significant effects were observed in average speed, F (6,397) = 1.965, p =.070, η²=.029, 95%CI (0.000, 0.055) and speed threshold F (6,397) =1.390, p=.217, η²=.021, 95%CI (0.000, 0.041).( Lines 199-203, Page 8)

Experiment 2：The results regarding peak speed showed a significant main effect of intervention, F (1,121) = 32.631, p<.001, 95%CI (0.092,0.327), with a large effect size (η²=0.212). The improvement in peak speed achieved by the intervention group was found to be significantly higher than that of the control group (0.84±0.08 vs. 0.22±0.08, p<.001, 95%CI [0.643,1.396]), with a large effect size (d=1.019). The result indicates that 3D-MOT training had a significant effect on improving the peak speed of young soccer players.The main effect of age was not significant, F (2,121) = 0.422, p=.657, η²=0.005, 95%CI (0.000,0.004). The interaction effect between group and age was not significant, F (2,121)=1.989, p=.141,η²=0.025, 95%CI (0.000,0.094).

The results revealed a significant main effect of intervention on average speed, F (1,121)=38.964, p<.001, 95%CI(0.153,0.399), with a large effect size (η²=.278). The improvement in average speed of the intervention group was significantly higher than that of the control group (0.68±0.06 vs.0.16±0.05, p <.001), with a large effect size (d=1.234). The 3D-MOT training significantly improved the average speed of young soccer players.There was no significant age effect observed the interaction between age, F (1,121)=1.215, p=.300, η²=.014, 95%CI (0.000,0.070), and intervention was not significant, F (1,121)=0.325, p=.723, η²=.004, 95%CI (0.000,0.038).

The results regarding the speed threshold showed that there was a significant main effect of intervention, F (1,121) =38.941, p<.001, 95%CI (0.119,0.360), with a large effect size (η²=0.239). The improvement in speed threshold of the intervention group was found to be significantly higher than that of the control group (0.75±0.08 vs. 0.11±0.07, p<.001, 95%CI [0.733,1.494]), with a large effect size (d=1.113). The 3D-MOT training significantly improved the speed threshold of young soccer players. No significant age effect was observed and the interaction between age, F (1,121) =0.458, p=.634, η²=.006, 95%CI (0.000,0.046), and intervention was not significant, F (1,121) =1.127, p=.327, η²=.014, 95%CI (0.000,0.070).( Lines 286-311, Page 12-13)

Comment 8: *Moreover, full reference of SPSS should be described. Please revise;*

Response 8: We sincerely appreciate the valuable comment. The following is our response to this comment.

According to the comment of the reviewer, we modified the description of using the SPSS program for analysis in the data analysis section.

The revised part now reads：Data were analyzed using Statistical Package for the Social Sciences (SPSS) version 27 (IBM corp. Released, 2020). ( Lines 195-196, Page 8)

Comment 9：*authors mentioned hypothesis but this was not explained in introduction. Thus, please clarify hypotheses of the study*

Response 9: We sincerely appreciate the valuable comment. The following is our response to this comment.

According to the comment of the reviewers, we revised the introduction part.

The revised part now reads：This research anticipates that the performance of 3D-MOT will improve with age, but the growth plateaus after the age of 15.and the 3D-MOT training effect may be less pronounced after age 15 [22]. ( Lines 97-99, Page 5)

**Response to Reviewer 1：**

Comment 1: *Abstract: Lines 19-20, Page 2: Need explicit acknowledgment in the abstract that data from players younger than 12 is missing, limiting conclusions about potential sensitive periods.*

Response 1:We sincerely appreciate the valuable comment. The following is our response to this comment.

According to the reviewers' comment, in the abstract, we further explicitly acknowledged that the absence of data for players under the age of 12 limited the conclusion on potential sensitive periods.

The revised part now reads：“Given the lack of data for those players younger than 12, this restricts the inference of whether there are potential sensitive periods.”(Lines 21-22, Page 2 )

Comment 2: *Introduction：Lines 32-36, Page 4: Requires more direct explanation of how 3D-MOT specifically relates to soccer performance compared to traditional MOT tasks..*

Response 2:We sincerely appreciate the valuable comment. The following is our response to this comment.

According to the comment of the reviewers, we have modified this part of the content.

The revised part now reads: “3D-MOT technology is an extension of the traditional MOT paradigm, aiming to simulate more realistic and complex motion scenarios [6]. The traditional paradigm of Multiple Object Tracking (MOT) mainly focuses on the two-dimensional plane [7], while 3D-MOT technology introduces depth information, making the tracking task closer to the motion situations in real life [8]. In a soccer match, players have to quickly and accurately judge the positions and movement trajectories of the ball, teammates, and opponents in the three-dimensional space. Therefore, 3D-MOT technology provides an effective tool for evaluating the dynamic visual attention of soccer players in complex visual environments [9].”(Lines 36-44, Page 3)

Comment 3:*Introduction:Lines 60-67, Page 5:Needs better connection between previous 3D-MOT research findings and soccer-specific skills or performance measures.*

Response 3:We sincerely appreciate the valuable comment. The following is our response to this comment.

According to the comment of the reviewers, we added the relevant content.

The revised part now reads: “For instance, Ehmann et al. revealed that there was a significant positive correlation between the performance of the 360°environmental MOT task and the accuracy score in the 360°soccer passing task , as well as the defensive performance score in the small-field game [21].. Moreover, elite youth players demonstrated better tracking performance than sub-elite youth players [12]. ”(Lines 72-77, Page 4)

Comment 4:*Introduction: Lines 89-92, Page 7: The claim about dynamic visual attention influencing sport performance requires additional supporting evidence..*

Response 4:We sincerely appreciate the valuable comment. The following is our response to this comment.

According to the comment of the reviewers, we added evidence support in the introduction.

The revised part now reads:“Moreover, elite youth players demonstrated better tracking performance than sub-elite youth players [12] ”(Lines 76-77, Page 4)

Comment 5: *Methods - Experiment 1：Lines 113-116, Page 8: The imbalanced sample sizes across age groups (especially n=16 for 12-year-olds) requires additional statistical justification.*

Response 5:We sincerely appreciate the valuable comment. The following is our response to this comment.

According to the comment of the reviewers, we added additional statistical justification.

The revised part now reads：“Data were weighted by inverse sample sizes to balance age group contributions[27]”(Lines 191-192 , Page 8)

Reviewer#1: **SPECIFIC COMMENTS**

Comment 6: *Methods - Experiment 1: Lines 142-143, Page 9: Citation needed to support the claim about technique effectiveness in measuring dynamic attention in soccer players.*

Response 6:We sincerely appreciate the valuable comment. The following is our response to this comment.

According to thecomment of the reviewers, we added relevant citations.

The revised part now reads: “ The stimuli consisted of 10 yellow 3D spheres with a diameter of 27.94cm [24]. ”(Lines 138-139, Page 6)

Comment 7: *Methods - Experiment 1: Lines 159-165, Page 10: Need rationale for the specific parameters chosen in the adaptive procedure (30% increase, 20% decrease).*

Response 7:We sincerely appreciate the valuable comment. The following is our response to this comment.

According to the comment of the reviewers, we added the relevant content.

The revised part now reads: “This method estimates the X•0 by calculating the midpoint of each pair of running peak and valley values, which is referred to as the midpoint running estimate. This approach is simple, robust, efficient and has a low estimation bias [25].”(Lines 156-158, Page 7)

Comment 8: *Lines 183-184, Page 11: Experiment dates should appear earlier in the methods as part of the study design description..*

Response 8:We sincerely appreciate the valuable comment. The following is our response to this comment.

According to the reviewers' comment, we placed the experimental date in a more forward position in the methods section.

The revised part now reads: “.This study adopted a cross-sectional design, and the 3D-MOT task started on December 1, 2020, and ended on December 20, 2020 (01/12/2020-20/12/2020). All participants were tested individually in a quiet laboratory, with each participant completing the experiment independently. Before the experiment, participants were allowed to practice three times. Each participant was then asked to complete 30 trials of the 3D-MOT task, which was estimated to take 15 minutes per person. All participants followed the same standardized procedure and standing position to complete the trials. The first author of the paper was responsible for administering the tests according to the standardized instructions provided to the participants.”(Lines 120-128, Page 6)

Comment 9: *Results - Experiment 1：Lines 203-207, Page 12: Effect sizes should include standardized interpretations (small, medium, large) consistently throughout.*

Response 9: We sincerely appreciate the valuable comment. The following is our response to this comment.

Based on the comment of the reviewers, we added the relevant content.

The revised part now reads: “We interpreted the magnitude of the effect size as small ( η²≤0.01), medium (η²≤0.06), and large (η²≤0.14),and for Cohen’s d are small (d≤0.20); medium (d≤0.50) and large (d≤0.80)[27].”(Line191-194, Page 8)

Comment 10: *Results - Experiment 1：Lines 208-213, Page 13: Table 1 should include effect sizes and confidence intervals for all comparisons.*

Response 10:We sincerely appreciate the valuable comment. The following is our response to this comment.

Based on the reviewers' comment, we revised the table and added the effect size and confidence interval for all comparisons.

The revised part now reads:

“**Table 1 The descriptive statistics for all groups.**

|  |  | Peak speed | | Average speed | | Speed threshold | |
| --- | --- | --- | --- | --- | --- | --- | --- |
| Group  （*I*） | Group（*J）* | Mean difference  (*I-J*)  Std.Error | Sig. (d)&  95% *CI* | Mean difference  (*I-J*)  Std.Error | Sig. (d) &  95% *CI* | Mean difference  (*I-J*)  Std.Error | Sig. (d)&  95% *CI* |
| U12 | U13 | -0.088  (0.180) | 1.000(-0.137)  (-0.476,0.300) | -0.039  (0.134) | 1.000(-0.083)  (-0.448,0.370) | -0.033  (0.163) | 1.000(-0.057)  (-0.530,0.465) |
|  | U14 | -0.349  (0.184) | 0.193(-0.543)  (-0.756,0.059) | -0.212  (0.137) | 1.000(-0.450)  (-0.631,0.207) | -0.147  (0.167) | 1.000(-0.257)  (-0.657,0.363) |
|  | U15 | -0.442  (0.170) | 0.002(-0.688)  (-0.783,0.100) | -0.252  (0.126) | 0.982(-0.535)  (-0.638,0.134) | -0.229  (0.154) | 1.000(-0.399)  (-0.698,0.241) |
|  | U16 | -0.214  (0.177) | 1.000(-0.333)  (-0.588,0.159) | -0.123  (0.131) | 1.000(-0.261)  (-0.525,0.279) | -0.054  (0.160) | 1.000(-0.094)  (-0.543,0.435) |
|  | U17 | -0.313  (0.174) | 0.182(-0.487)  (-0.676,0.050) | -0.172  (0.130) | 1.000(-0.365)  (-0.568,0.225) | -0.106  (0.158) | 1.000(-0.185)  (-0.588,0.376) |
|  | U18 | -0.371  (0.186) | 0.137(-0.578)  (-0.786,0.044) | -0.251  (0.138) | 1.000(-0.534)  (-0.674,0.172) | -0.237  (0.168) | 1.000(-0.415)  (-0.752,0.277) |
| U13 | U14 | -0.261  (0.127) | 0.929(-0.406)  (-0.655,0.134) | -0.173  (0.095) | 1.000(-0.367)  (-0.462,0.117) | -0.114  (0.115) | 1.000(-0.200)  (-0.466,0.238) |
|  | U15 | -0.354  (0.105)**^*^** | 0.021(-0.550)  (-0.680,-0.027) | -0.213  (0.078) | 0.143(-0.452)  (-0.452,0.026) | -0.196  (0.095) | 0.842(-0.342)  (-0.487,0.095) |
|  | U16 | -0.126  (0.116) | 1.000(-0.196)  (-0.486,0.234) | -0.084  (0.086) | 1.000(-0.178)  (-0.348,0.180) | -0.021  (0.105) | 1.000(-0.037)  (-0.342,0.300) |
|  | U17 | -0.225  (0.112) | 1.000(-0.350)  (-0.573,0.124) | -0.133  (0.084) | 1.000(-0.282)  (-0.388,0.123) | -0.074  (0.102) | 1.000(-0.128)  (-0.384,0.237) |
|  | U18 | -0.283  (0.130) | 0.677(-0.440)  (-0.685,0.119) | -0.212  (0.096) | 1.000(-0.451)  (-0.507,0.083) | -0.205  (0.117) | 1.000(-0.358)  (-0.564,0.154) |
| U14 | U15 | -0.093  (0.113) | 1.000(-0.145)  -0.442,0.256) | -0.040  (0.084) | 1.000(-0.085)  (-0.296,0.216) | -0.082  (0.102) | 1.000(-0.143)  (-0.393,0.230) |
|  | U16 | 0.135  (0.123) | 1.000(-0.210)  (-0.246,0.515) | 0.089  (0.091) | 1.000(0.189)  (-0.190,0.368) | 0.093  (0.111) | 1.000(0.163)  (-0.246,0.433) |
|  | U17 | 0.036  (0.119) | 1.000(-0.056)  (-0.334,0.406) | 0.04  (0.089) | 1.000(0.085)  (-0.231,0.312) | 0.041  (0.108) | 1.000(0.071)  (-0.289,0.371) |
|  | U18 | -0.022  (0.136) | 1.000(-0.035)  (-0.444,0.399) | -0.039  (101) | 1.000(-0.084)  (-0.348,0.270) | -0.090  (0.123) | 1.000(-0.158)  (-0.466,0.285) |
| U15 | U16 | 0.228  (0.1) | 0.525(-0.354)  (-0.082,0.537) | 0.129  (0.074) | 1.000(0.274)  (-0.098,0.356) | 0.175  (0.090) | 1.000(0.305)  (-0.101,0.451) |
|  | U17 | 0.129  (0.095) | 1.000(-0.201)  (-0.167,0.425) | 0.08  (0.071) | 1.000(0.170)  (-0.137,0.297) | 0.123  (0.086) | 1.000(0.214)  (-0.142,0.387) |
|  | U18 | 0.071  (0.115) | 1.000(-0.110)  (-0.287,0.429) | 0.001  (0.086) | 1.000(0.001)  (-0.262,0.263) | -0.009  (0.104) | 1.000(-0.015)  (-0.328,0.311) |
| U16 | U17 | -0.099  (0.107) | 1.000(-0.154)  (-0.432,0.234) | -0.049  (0.080) | 1.000(-0.103)  (-0.293,0.195) | -0.052  (0.097) | 1.000(-0.091)  (-0.349,0.244) |
|  | U18 | -0.157  (0.125) | 1.000(-0.244)  (-0.546,0.232) | -0.128  (0.093) | 1.000(-0.272)  (-0.413,0.157) | -0.184  (0.113) | 1.000(-0.321)  (-0.530,0.163) |
| U17 | U18 | -0.058  (0.122) | 1.000(-0.090)  (-0.436,0.320) | -0.08  (0.091) | 1.000(-0.169)  (-0.457,0.198) | -0.131  (0.110) | 1.000(-0.229)  (-0.469,0.206) |

”(Lines 206, Page 8-9 )

Comment 11: *Results - Experiment 1：Lines 216-221, Page 14: Confidence intervals for the significant difference between 13 and 15-year-olds need consistency with other statistical reporting.*

Response 11: We sincerely appreciate the valuable comment. The following is our response to this comment.

Based on the comment of the reviewers, we added the relevant content.

The revised part now reads: “Further pairwise analysis using Bonferroni's post hoc test indicated that the peak speed of the 13-year-old group was significantly lower than that of the 15-year-old group (p =. 018, 95%CI [-0.675,-0.032]), with a medium effect size (d=-0.559). ”(Line213-215, Page 10)

Reviewer#1: **SPECIFIC COMMENTS**

Comment 12: *Results - Experiment 2：Lines 236-248, Page 15: Need justification for the large effect size (.80) expected in the power analysis based on previous research.*

Response 12:We sincerely appreciate the valuable comment. The following is our response to this comment.

According to the reviewers' comment, we modified this part.

The revised part now reads:“The sample size for this study was determined by estimating the effect size f using the G*power software 3.1.9.7[28]. This study set the computation for a 2 ×3 analysis of variance (ANOVA) and set the expected effect size f at 0.25, theαlevel was set at 0.05, the desired power (1−β) at 80%, and the correlation between repeated measures at 0.5. ”(Lines 236-240, Page 11)

*Comment 13*:*Results - Experiment 2： Lines 252-255, Page 15: The short intervention period (15 sessions over 5 days) requires stronger justification.*

Response 13:We sincerely appreciate the valuable comment. The following is our response to this comment.

Based on the reviewers' comment, we added relevant citations to prove the effectiveness of the short-term intervention.

The revised part now reads: “Short-term intervention has been proven to effectively improve 3D-MOT performance in previous studies. Therefore, the short-term training program in previous studies was followed in this study. The specific plan is as follows：

The intervention group received 15 sessions of active training for five consecutive days from December 23rd to December 28th, 2020 (24/12/2020-28/12/2020). They participated in three sessions of 3D-MOT task a day, of which, one session includes 30 trials [9]. ”(Lines 255-256, Page 11 )

Reviewer#1: **SPECIFIC COMMENTS**

*Comment 14*:*Results - Experiment 2：Lines 274-276, Page 17: Figure 4 is referenced but not visible in the manuscript.*

Response 14:We sincerely appreciate the valuable comment. The following is our response to this comment.

Based on the reviewers' comment, we examined Figure 4 and ensured that it was uploaded to the system.

*Comment 15*: *Results - Experiment 2：Lines 283-288, Page 17: Include interpretation of the effect size (η²=0.212) for the main effect of intervention on peak speed.*

Response 15:We sincerely appreciate the valuable comment. The following is our response to this comment.

According to the comment of the reviewers, we added this part.

The revised part now reads: “The result indicates that 3D-MOT training had a significant effect on improving the peak speed of young soccer players. ”(Lines 299-300, Page 13 )

*Comment 16*:*Results - Experiment 2：* *Lines 289-293, Page 17: Need interpretation of practical significance for average speed improvements..*

Response 16:We sincerely appreciate the valuable comment. The following is our response to this comment.

According to the comment of the reviewers, we added this part.

The revised part now reads: “The 3D-MOT training significantly improved the average speed of young soccer players.”(Lines 290-292, Page 12 )

Comment 17: *Discussion:Lines 328-333, Page 19: The speculation about development peaks before age 12 lacks direct supporting data from this study and requires clearer acknowledgment of this limitation.*

Response 17:We sincerely appreciate the valuable comment. The following is our response to this comment.

Based on the reviewers' comments, we further clarified the limitations of this part during the discussion.

The revised part now reads: “Given the lack of sample data of those under the age of 12 in this study, it has to some extent limited the inference of this possibility.Future research could explore the MOT development in younger children to provide a more comprehensive understanding of the age-related changes in MOT performance.” (Lines341-345, Page 14)

Comment 18: *Discussion:Lines 339-353, Page 20: Expand the discussion of peak speed sensitivity to include specific implications for training and assessment protocols.*

Response 18:We sincerely appreciate the valuable comment. The following is our response to this comment.

According to the reviewers' comment, we added some relevant content in the discussion.

The revised part now reads: “At present, there are mainly three computational methods for studying the tracking performance of 3D-MOT tasks. Firstly, the fastest speed among all the correct trials is taken as the peak speed representing the individual's tracking ability[10]. Secondly, calculate the overall average of all trial reversals within the experiment as the average threshold to represent the individual's tracking ability[26]. Finally, based on the assumption that an individual's responses tend to stabilize in the later stage of the experiment, the average of the last four reversals of the task is taken (i.e., speed threshold) as the individual's tracking ability[8].” (Lines353-360, Page 14-15)

Comment 19: *Discussion: Lines 354-376, Page 21: Neural mechanisms discussion needs more direct connection to the study's findings.*

Response 19:We sincerely appreciate the valuable comment. The following is our response to this comment.

According to the reviewers' comment, we modified this part during the discussion and deleted the discussion related to the neural mechanism.

The revised part now reads: “These findings suggest that regular training in 3D-MOT tasks may have a positive impact on the development of these cognitive skills in young soccer players. It is plausible that the improvements observed in Experiment 2 were due to the specific training regime that focused on enhancing MOT abilities [36]. This highlights the potential of targeted training interventions in facilitating the cognitive development of young players.” (Lines384-389, Page 15)

Comment 20: *Discussion: Lines 377-387, Page 22: More clearly identify the speculative nature of sensitive period discussions given the study design limitations.*

Response 20:We sincerely appreciate the valuable comment. The following is our response to this comment.

Based on the reviewers' comment, we have revised this part during the discussion and further emphasized the limitations of this study

The revised part now reads: “During this stage, the myelination process of the prefrontal-parietal network accelerates [39], and synaptic pruning leads to an improvement in neural efficiency [40], which may make the brain particularly sensitive to adaptive training in the allocation of attentional resources. Thus, initiating 3D-MOT training earlier in this sensitive period may yield more pronounced improvements in cognitive abilities. In view of the limitations of the research design of this study, future research can consider including younger participants, such as children aged 8-12, to explore whether there is a specific developmental “window period” for 3D-MOT training.” (Lines399-406, Page 16)

Comment 21: *Limitations and Future Directions:Lines 413-414, Page 24: Include specific recommendations for minimum sample sizes in future research based on power analyses.*

Response 21:We sincerely appreciate the valuable comment. The following is our response to this comment.

Based on the reviewers' comment, our specific suggestions for the minimum sample size.

The revised part now reads: “Future research is recommended to recruit at least 33 participants to ensure a minimum statistical effect size, thereby enhancing the credibility of the research results. ” (Lines419-421,Page 16 )

Comment 22: *Conclusion: Lines 416-426, Page 24: Address practical implications for coaches and training programs more explicitly.*

Response 22:We sincerely appreciate the valuable comment. The following is our response to this comment.

Based on the reviewers' comments, we elaborated more clearly on the application of practice in the conclusion.

The revised part now reads: “These results provide valuable references for coaches, helping them understand the development of cognitive abilities of young soccer players and design diversified training plans that include cognitive stimulation to effectively enhance the cognitive abilities of players.” (Lines432-435,Page 17 )

We tried our best to improve the manuscript and made some changes marked in red in revised paper which will not influence the content and framework of the paper. We appreciate for Editors and Reviewers' warm work earnestly, and hope the correction will meet with approval. Best regards!
