# Supplementary material for: Three-dimensional multiple object tracking (3D-MOT) performance in young soccer players: Age-related development and training effectiveness
Source: PLoS One. 2025 Aug 1;20(8):e0312051. doi: 10.1371/journal.pone.0312051 (PMC12316292; doi:10.1371/journal.pone.0312051)
Supplement: Peer review history

# Peer review: Decision Letter 0

**DOI:** 10.1371/journal.pone.0312051.r001

**Authors:** Rafael Oliveira

Dear Dr. <!--StartFragment-->Zhang,

Thank you for submitting your manuscript to PLOS ONE. After careful consideration, we feel that it has merit but does not fully meet PLOS ONE’s publication criteria as it currently stands. Therefore, we invite you to submit a revised version of the manuscript that addresses the points raised during the review process.

Please submit your revised manuscript by Feb 21 2025 11:59PM. If you will need more time than this to complete your revisions, please reply to this message or contact the journal office at plosone@plos.org . When you're ready to submit your revision, log on to https://www.editorialmanager.com/pone/ and select the 'Submissions Needing Revision' folder to locate your manuscript file.

- A rebuttal letter that responds to each point raised by the academic editor and reviewer(s). You should upload this letter as a separate file labeled 'Response to Reviewers'.
- A marked-up copy of your manuscript that highlights changes made to the original version. You should upload this as a separate file labeled 'Revised Manuscript with Track Changes'.
- An unmarked version of your revised paper without tracked changes. You should upload this as a separate file labeled 'Manuscript'.

If you would like to make changes to your financial disclosure, please include your updated statement in your cover letter. Guidelines for resubmitting your figure files are available below the reviewer comments at the end of this letter.

If applicable, we recommend that you deposit your laboratory protocols in protocols.io to enhance the reproducibility of your results. Protocols.io assigns your protocol its own identifier (DOI) so that it can be cited independently in the future. For instructions see: https://journals.plos.org/plosone/s/submission-guidelines#loc-laboratory-protocols . Additionally, PLOS ONE offers an option for publishing peer-reviewed Lab Protocol articles, which describe protocols hosted on protocols.io. Read more information on sharing protocols at https://plos.org/protocols?utm_medium=editorial-email&utm_source=authorletters&utm_campaign=protocols .

We look forward to receiving your revised manuscript.

Kind regards,

Rafael Oliveira

Academic Editor

PLOS ONE

Journal Requirements:

When submitting your revision, we need you to address these additional requirements.

1. Please ensure that your manuscript meets PLOS ONE's style requirements, including those for file naming. The PLOS ONE style templates can be found at 

https://journals.plos.org/plosone/s/file?id=wjVg/PLOSOne_formatting_sample_main_body.pdf and 

https://journals.plos.org/plosone/s/file?id=ba62/PLOSOne_formatting_sample_title_authors_affiliations.pdf

2. Thank you for stating the following financial disclosure: The study was supported by National Natural Science Foundation of China (No.32071087).   

Please state what role the funders took in the study.  If the funders had no role, please state: ""The funders had no role in study design, data collection and analysis, decision to publish, or preparation of the manuscript."" 

If this statement is not correct you must amend it as needed. 

Please include this amended Role of Funder statement in your cover letter; we will change the online submission form on your behalf.

3. Please include captions for your Supporting Information files at the end of your manuscript, and update any in-text citations to match accordingly. Please see our Supporting Information guidelines for more information: http://journals.plos.org/plosone/s/supporting-information. 

Additional Editor Comments:

Dear authors,

This manuscript has potential to be published. Indeed, one reviewer provided a good opinion. However, reviewer 1 stated otherwise. The manuscruot addresses an important topic but the current form has significant flaws in the study design and analysis that limit its current contribution. With major revisions to address the sample size, control group, statistical approach, and depth of discussion, it could become a valuable addition to the literature. However, the authors will need to conduct additional analyses and potentially collect more data to adequately address these concerns.

Therefore, major revisions are required.

Thank you

[Note: HTML markup is below. Please do not edit.]

Reviewers' comments:

Reviewer's Responses to Questions

**Comments to the Author**

1. Is the manuscript technically sound, and do the data support the conclusions?

Reviewer #1: Yes

Reviewer #2: Yes

**********

2. Has the statistical analysis been performed appropriately and rigorously?

Reviewer #1: Yes

Reviewer #2: Yes

**********

3. Have the authors made all data underlying the findings in their manuscript fully available?

The PLOS Data policy

Reviewer #1: No

Reviewer #2: Yes

**********

4. Is the manuscript presented in an intelligible fashion and written in standard English?

Reviewer #1: Yes

Reviewer #2: Yes

**********

Reviewer #1: General Comments:

This study examines the effects of a 12-week high-intensity interval training program on physical fitness parameters in youth soccer players. The topic is relevant and timely given the growing interest in optimizing training methods for young athletes. However, there are several major and minor issues that need to be addressed before the manuscript can be considered for publication.

Major weaknesses:

1. The small sample size (n=20) limits the statistical power and generalizability of the results. A power analysis should be conducted to determine if the sample size was adequate.

2. The control group received no training intervention, which does not allow for comparison to a standard training program. A more appropriate control would be a group following a traditional soccer training regimen.

3. The statistical analysis is inadequate. Only paired t-tests were used to assess pre-post changes within groups. Between-group comparisons and an analysis of the interaction effects (e.g. ANOVA) are needed.

4. The discussion lacks depth in interpreting the results in the context of existing literature. More critical analysis of the findings and their implications is required.

Minor weaknesses:

1. Some methodological details are missing, such as familiarization procedures and reliability of the fitness tests.

2. The results section needs reorganization for clarity and completeness.

3. There are numerous grammatical and typographical errors throughout.

Specific Comments:

Title: Consider revising to more accurately reflect the specific population studied (e.g., "Effects of High-Intensity Interval Training on Physical Fitness in Youth Soccer Players: A 12-Week Intervention Study").

Abstract:

Background:

Line 5: Specify the age range of "youth" soccer players included in this study.

Line 7: Briefly mention why HIIT might be particularly beneficial for soccer players.

Objective:

Line 10: Clarify if there were any secondary objectives beyond assessing physical fitness parameters.

Methods:

Line 15: State the exact number of participants in each group (HIIT and control).

Line 17: Provide brief details on the HIIT protocol (frequency, duration, work:rest ratio).

Line 20: List the specific physical fitness parameters measured.

Line 22: Mention the timing of assessments (pre- and post-intervention).

Results:

Line 25: Include actual values (mean ± SD) for pre- and post-intervention measures, not just p-values.

Line 28: Report effect sizes in addition to statistical significance.

Line 30: Mention any findings related to the control group.

Conclusion:

Line 35: Temper the conclusion to better reflect the limitations of the study design.

Line 38: Include a brief statement on the practical implications of the findings.

Keywords:

Add 1-2 additional relevant keywords (e.g., "adolescent athletes", "soccer-specific fitness").

Introduction:

Page 1, line 15: Provide more recent statistics on youth soccer participation rates to emphasize the relevance of the study.

Page 1, line 20: Define "high-intensity interval training" (HIIT) more precisely, including typical work-to-rest ratios and intensities used in soccer contexts.

Page 1, line 25: Elaborate on the physiological demands of soccer, particularly for youth players. Cite relevant studies on energy system utilization during match play.

Page 1, line 30: Discuss the current understanding of fitness requirements for successful performance in youth soccer. Reference position-specific demands if applicable.

Page 2, line 35: Provide a brief overview of traditional training methods used in youth soccer and their limitations.

Page 2, line 40: Explain the potential advantages of HIIT for youth soccer players, such as time efficiency or greater physiological adaptations.

Page 2, line 45: Summarize previous research on HIIT in adult soccer players, highlighting the gap in knowledge for youth populations.

Page 2, line 50: Discuss any existing studies on HIIT in youth soccer, noting their findings and limitations.

Page 2, line 55: Address potential concerns or controversies surrounding high-intensity training in youth athletes (e.g., injury risk, burnout).

Page 2, line 60: Explain the importance of studying different fitness parameters (aerobic capacity, sprint ability, agility) in the context of soccer performance.

Page 2, line 65: Discuss the rationale for the 12-week intervention duration chosen for this study.

Page 2, line 70: Provide a clearer justification for why this particular study is necessary and how it adds to the existing body of knowledge.

Page 2, line 75: Elaborate on the potential practical implications of the study findings for coaches and sports scientists working with youth soccer players.

Page 2, line 80: Consider addressing how growth and maturation factors might influence training adaptations in youth players.

Page 2, line 85: Clearly state the primary and secondary objectives of the study.

Page 2, line 90: Formulate specific, testable hypotheses based on the existing literature and the study objectives.

Methods:

Page 3, line 65: Specify the exact age range of participants, not just "youth players".

Page 3, line 68: Provide details on how medical clearance was obtained for participants.

Page 3, line 70: State whether informed consent was obtained from parents/guardians for underage participants.

Page 3, line 72: Clarify how participants were recruited and allocated to groups. Was randomization used?

Page 3, line 75: Clarify if participants were from a single soccer club or multiple organizations.

Page 3, line 78: Describe any inclusion/exclusion criteria used for participant selection.

Page 3, line 80: Provide more details on the HIIT protocol - work:rest ratios, total session duration, etc.

Page 3, line 82: Provide rationale for the 12-week intervention duration.

Page 3, line 85: Specify the exact frequency of HIIT sessions per week.

Page 3, line 88: Detail the warm-up and cool-down protocols used before and after HIIT sessions.

Page 4, line 92: Explain how exercise intensity was monitored and controlled during HIIT (e.g. heart rate, RPE).

Page 4, line 95: Describe any nutritional guidelines or restrictions given to participants.

Page 4, lines 95-100: Were the testers blinded to group allocation? How was test-retest reliability established?

Page 4, line 98: Provide information on participant adherence and any dropouts during the study.

Page 4, line 102: Specify the time of day when fitness tests were conducted and if this was standardized.

Page 4, line 105: Detail the recovery time allowed between different fitness tests.

Page 4, line 108: Explain how the order of fitness tests was determined and if this was randomized.

Page 4, line 110: Justify the use of only paired t-tests for statistical analysis. Consider using more robust methods.

Page 4, line 112: Provide manufacturer details for any equipment used in fitness assessments.

Page 4, line 115: Describe any familiarization sessions conducted prior to baseline testing.

Page 4, line 118: Specify the number of trials allowed for each fitness test and how the final score was determined.

Page 4, line 122: Detail any reliability assessments conducted for the fitness tests used.

Page 4, line 125: Explain how potential confounding variables (e.g. outside physical activity) were controlled or measured.

Results:

Page 5, Table 1: Include effect sizes in addition to p-values.

Page 5, lines 130-135: Present the between-group comparisons, not just within-group changes.

Page 6, Figure 1: Error bars should be added to the graphs.

Discussion:

Page 7, lines 180-185: Discuss potential mechanisms underlying the observed improvements in fitness.

Page 7, line 182: Expand on how the observed improvements in VO2max compare to other HIIT studies in youth soccer players. Cite relevant literature.

Page 7, line 190: Discuss potential physiological mechanisms underlying the improvements in aerobic capacity. Consider referencing studies on cardiac adaptations to HIIT in adolescents.

Page 7, line 198: Address the lack of significant improvement in sprint performance. Compare this finding to other research and propose possible explanations.

Page 7, line 205: Elaborate on the practical implications of the agility improvements. How might these translate to on-field performance?

Page 8, line 210: Critically analyze the magnitude of changes observed. Are these improvements clinically meaningful for youth soccer players?

Page 8, lines 210-215: Address the limitations of the study design and statistical approach more thoroughly.

Page 8, line 218: Discuss how the HIIT protocol used in this study compares to traditional soccer training methods in terms of time efficiency and effectiveness.

Page 8, lines 225-230: Provide more specific recommendations for future research and practical applications.

Page 8, line 225: Address the potential long-term effects of HIIT on youth athletes. Consider discussing any relevant literature on growth and maturation.

Page 8, line 232: Explore possible gender differences in response to HIIT, given that both male and female players were included in the study.

Page 8, line 240: Discuss how the findings might apply to different playing positions in soccer, as positional demands vary.

Page 8, line 248: Address the potential limitations of implementing HIIT in youth soccer, such as motivation, adherence, or injury risk.

Page 9, line 255: Explore how these findings might inform periodization strategies for youth soccer training programs.

Page 9, line 262: Discuss any observed or potential differences in individual responses to the HIIT program among participants.

Page 9, line 270: Address the ecological validity of the study - how well does the HIIT protocol mimic the demands of soccer match play?

Page 9, line 278: Elaborate on future research directions, suggesting specific study designs or variables to investigate further.

Page 9, line 285: Discuss potential psychological effects of HIIT on youth athletes, such as enjoyment or perceived exertion compared to traditional training.

Page 9, line 292: Address how these findings might apply to other youth team sports beyond soccer.

Insert the bellow references in the discussion section:

Dhahbi, W., Zouita, A., Salah, F. B., Chaouachi, A., Chamari, K., & Cheze, L. (2014). Reference database of the gait cycle for young healthy Tunisian adults. IRBM, 35(1), 46-52.

Dhahbi, W., Chaouachi, A., Cochrane, J., Chèze, L., & Chamari, K. (2017). Methodological issues associated with the use of force plates when assessing push-ups power. The Journal of Strength & Conditioning Research, 31(7), e74.

Conclusion:

Page 9, lines 245-250: The conclusion overstates the findings given the study limitations. Temper the language.

Reviewer #2: Thank you for the opportunity to review this manuscript, which explores the age-related development and training effectiveness of three-dimensional multiple object tracking (3D-MOT) performance in young soccer players. The study design is well-conceived and demonstrates practical applications that can be effectively implemented in real-world training scenarios. While I believe the manuscript has strong potential for acceptance

**********

what does this mean? ). If published, this will include your full peer review and any attached files.

If you choose “no”, your identity will remain anonymous but your review may still be made public.

**Do you want your identity to be public for this peer review?** For information about this choice, including consent withdrawal, please see our Privacy Policy

Reviewer #1: **Yes: ** Wissem Dhahbi

Reviewer #2: No

**********

[NOTE: If reviewer comments were submitted as an attachment file, they will be attached to this email and accessible via the submission site. Please log into your account, locate the manuscript record, and check for the action link "View Attachments". If this link does not appear, there are no attachment files.]

While revising your submission, please upload your figure files to the Preflight Analysis and Conversion Engine (PACE) digital diagnostic tool, https://pacev2.apexcovantage.com/ . PACE helps ensure that figures meet PLOS requirements. To use PACE, you must first register as a user. Registration is free. Then, login and navigate to the UPLOAD tab, where you will find detailed instructions on how to use the tool. If you encounter any issues or have any questions when using PACE, please email PLOS at figures@plos.org . Please note that Supporting Information files do not need this step.

# Author comment: Author response to Decision Letter 1

**DOI:** 10.1371/journal.pone.0312051.r002

26 Apr 2025

Response to the Reviewer's Comments

Manuscript number�PONE-D-24-37901

Title:

Three-dimensional multiple object tracking (3D-MOT) performance in young soccer players: age-related development and training effectiveness

Dear Editors and Reviewers,

We appreciate the opportunity to revise our manuscript titled "Three-dimensional multiple object tracking (3D-MOT) performance in young soccer players: age-related development and training effectiveness". We are grateful for the insightful comments provided by the reviewers. Those comments are all valuable and very helpful for revising and improving our paper, as well as the important guiding significance to our researches. In the following, we have provided detailed responses to each of the reviewers' comments. Revised portion are marked in red in the paper. Additionally, we have conducted a comprehensive revision of the entire manuscript. In this response letter, the reviewers' comments are presented in italics, and our corresponding changes and additions to the manuscript are highlighted in red text. We have tried our best to make all the revisions clear, and we hope that the revised manuscript meets the requirements for publication.

Reviewer#1: Major Weaknesses

Comment 1: Lack of Pre-12-Year-Old Data: The study focuses on players aged 12-18, but the absence of data from players younger than 12 limits the ability to identify potential sensitive periods for 3D-MOT development. This is a significant gap, as the authors suggest that rapid growth in MOT ability may occur before age 12 (Lines 306-311, Page 17).

Response 1: First of all, we would like to express our sincere gratitude to the reviewer for your insightful comments and valuable feedback. The following is our response to this comment.

At the beginning of the research design, numerous literatures have indicated that the maturation process of the adolescent brain continues until the end of adolescence. For instance, the changes in the ratio of white matter to gray matter in the brain suggest that the brain structure still undergoes large-scale remodeling in late adolescence. In light of this, this study mainly delves into the age-related perceptual-cognitive ability changes of athletes from early adolescence to early adulthood. As the results did not meet our expectations, the hypothesis that the rapid growth of MOT ability may occur before the age of 12 was proposed in the discussion. Regarding this major limitation, we suggested in the research outlook section that future studies should focus on the cognitive ability development of athletes before the age of 12.

Reviewer#1: Major Weaknesses

Comment 2: Small Sample Size for Younger Age Groups: The sample size for the 12-year-old group is notably small (n=16), which may affect the generalizability of the findings (Lines 91-94, Page 6).

Response 2: We sincerely appreciate the valuable comment. The following is our response to this comment.

As the experiment was conducted during the COVID-19 pandemic, many players in the 12-year-old group were unable to participate, which to some extent limited the sample size. In response to this comment, we have included a discussion in our study on the impact of the relatively small sample size in the 12-year-old group on the research results.

The revised part now reads�“Notably, no significant intergroup differences were observed between the age 12 group and other age groups. This might be attributed to limited statistical power resulting from the comparatively smaller sample size within the age 12 group (n = 16). Future studies could further expand the sample size, especially for the age group 12, to verify this hypothesis. ” (Lines 339-343, Page 19,)

Reviewer#1: Major Weaknesses

Comment 3: Limited Exploration of Transfer Effects: The study does not explore whether improvements in 3D-MOT performance transfer to actual soccer performance on the field. This limits the practical implications of the findings (Lines 329-352, Pages 19-20).

Response 3: We sincerely appreciate the valuable comment. The following is our response to this comment.

The core objective of this study is to explore whether the improvement of players' cognitive abilities is associated with specific sensitive periods of age growth, rather than the transfer effect of training. In response to this comment, we have added in the discussion that future research should explore the transfer impact of 3D-MOT training on actual soccer performance.

Reviewer#1: Major Weaknesses

Comment 4:Insufficient Discussion on Mechanisms: The paper lacks a detailed discussion on the underlying mechanisms that drive the observed improvements in 3D-MOT performance, particularly in relation to cognitive and neural development during adolescence.

Response 4:We sincerely appreciate the valuable comment. The following is our response to this comment.

In response to this comment, we have added a discussion on the potential neural mechanisms underlying the improvement of 3D-MOT performance in our deliberations, and analyzed the connection between the cognitive and neural development processes during adolescence and the enhanced training effects of 3D-MOT.

The revised part now reads:“Previous research evidence has revealed the significant role of three-dimensional multi-object tracking (3D-MOT) in enhancing attention, visual information processing speed, and working memory[42,43].The brain imaging studies have further elucidated the neural mechanisms underlying the enhancement of cognitive abilities by 3D-MOT, particularly in the parietal and frontal lobes of the cerebral cortex, which are considered key regions for attention shifts and eye movement control[44].These brain pathways may play a role in football players' acquisition of game information during matches. In this sense, during the 3D-MOT process, the attentional tracking of multiple elements may overlap with the brain networks required to obtain key information during the player's competition. By training these brain networks, soccer players might be able to more effectively filter and track key information during the game, thereby making quick and accurate responses. In the future, it is necessary to combine image or electroencephalogram research to explore the transfer effect of 3D-MOT performance and the neural processes behind the improvement.( Lines 371-385, Page 21-22)

…… During this stage, the myelination process of the prefrontal-parietal network accelerates[46], and synaptic pruning leads to an improvement in neural efficiency[47], which may make the brain particularly sensitive to adaptive training in the allocation of attentional resources. Notably, the functional connectivity between the anterior cingulate cortex and the dorsolateral prefrontal cortex gradually matures during this period[48], facilitating more effective cognitive control and attention regulation. Thus, initiating 3D-MOT training earlier in this sensitive period may yield more pronounced improvements in cognitive abilities. ”( Lines 389-396, Page 22-23)

Reviewer#1: Minor Weaknesses:

Comment 1: Repetition in Discussion: Some points in the discussion are repetitive, particularly regarding the flat developmental trajectory and the lack of interaction between age and training effects (Lines 287-289, Page 16; Lines 374-377, Page 21).

Response 1: We sincerely appreciate the valuable comment. The following is our response to this comment.

We have deleted the repetitive content in the discussion.

Comment 2: Clarification Needed on Speed Measurements: While the study uses peak speed, average speed, and speed threshold, the rationale for selecting these specific metrics and their sensitivity to age-related changes could be better explained (Lines 143-153, Page 9).

Response 2: We sincerely appreciate the valuable comment. The following is our response to this comment.

We have added the missing content raised in the comments (the analysis of the basic principles of specific indicators and their sensitivity to age-related changes) in the discussion.

The revised part now reads�“It is noteworthy that the results of this study found that the peak tracking speed seems to be more sensitive to changes in athletes' age than the average speed and the speed threshold. The present study examined age-related changes in task performance using the three dependent variables and found that peak speed was more sensitive to age-related development than the other measurements. Peak speed represents the highest velocity that a player can track as the target object progressively accelerates. The sensitivity of peak speed to age-related changes may be attributed to its direct reflection of an individual's maximum capacity and efficiency in processing visual information. As an indicator of an player's ability to rapidly and accurately track multiple objects, peak speed highlights the cognitive and perceptual limits under high-demand conditions[10]. In contrast, average speed encompasses the speed of all object movements throughout the entire task, which may mask age-related differences that are more pronounced at the performance ceiling[37]. Similarly, the speed threshold, which is the average speed of the last four reversals of the object, may be influenced by factors unrelated to age, such as differences in individual task engagement, thereby attenuating age-related effects [17]. In sum, this provides novel evidence for future researchers to utilize the 3D-MOT task as an assessment tool. ”( Lines 347-363, Page 20-21)

Comment 3: Cl Statistical Reporting: The statistical reporting could be more detailed, particularly in terms of effect sizes and confidence intervals, to provide a clearer understanding of the magnitude of the observed effects.

Response 3: We sincerely appreciate the valuable comment. The following is our response to this comment.

In response to this comment, we have added confidence intervals in the results section. Given that we employed the analysis of variance method, the ŋp2 index reflects the magnitude of the effect size.

The revised part now reads�

Group 3D-MOT performance

Speed Peak M(SD) 95% CI Avg M(SD) 95% CI ST M(SD) 95% CI

U12 1.34 (0.78) 1.03,1.65 1.01 (0.65) 0.78,1.25 1.22 (0.80) 0.94,1.50

U 13 1.43 (0.71)a* 1.26,1.60 1.05 (0.50) 0.93,1.18 1.25 (0.66) 1.10,1.41

U 14 1.69 (0.60) 1.51,1.88 1.23 (0.46) 1.09,1.37 1.37 (0.55) 1.20,1.54

U 15 1.78 (0.67)a* 1.66,1.91 1.27 (0.49) 1.18,1.36 1.45(0.58) 1.34,1.56

U 16 1.56 (0.61) 1.40,1.71 1.14 (0.44) 1.02,1.25 1.28 (0.54) 1.14,1.42

U 17 1.66 (0.56) 1.51,1.80 1.19 (0.43) 1.08,1.29 1.33 (0.52) 1.20,1.46

U 18 1.71 (0.52) 1.52,1.91 1.27 (0.43) 1.12,1.41 1.46 (0.47) 1.29,1.63

“Further pairwise analysis using Bonferroni's post hoc test indicated that the peak speed of the 13-year-old group was significantly lower than that of the 15-year-old group (1.43vs 1.78, p =.018, 95%CI [-0.68,-0.32]).” (Lines 215[Table 1] and 225,Page 20-21)

Reviewer#1: SPECIFIC COMMENTS

Comment 1: Abstract: The abstract provides a good summary of the study's objectives, methods, results, and conclusions. However, it could briefly mention the lack of data for players younger than 12 as a limitation (Lines 1-20, Page 2).

Response 1:We sincerely appreciate the valuable comment. The following is our response to this comment.

The abstract section supplements the limitations of this study, namely, the data of players under the age of 12 were not included.

The revised part now reads�“We suggest that in the future, more evidence on athletes under the age of 12 be added to enhance the understanding of the full developmental trajectory of 3D-MOT performance in soccer players. ”(Lines 21-23, Page 3 )

Reviewer#1: SPECIFIC COMMENTS

Comment 2:Introduction: Lines 22-26, Page 3: The introduction effectively sets the stage by highlighting the importance of dynamic visual attention in soccer. However, it could benefit from a more detailed discussion of how 3D-MOT specifically relates to soccer performance.

Response 2:We sincerely appreciate the valuable comment. The following is our response to this comment.

Regarding this comment, we have added the relevant content in the first paragraph.

The revised part now reads: “For example, during a game, players have to simultaneously focus on multiple dynamic information within a short period of time, including the trajectory of the ball, the positions of their opponents, and the movements of their teammates.This information is crucial for players to make reasonable decisions. ”(Page 4, Lines 29-32)

Reviewer#1: SPECIFIC COMMENTS

Comment 3:Introduction: Lines 40-56, Page 4: The discussion of 3D-MOT technology and its application in sports is well-presented. However, the authors could provide more context on how 3D-MOT differs from traditional MOT tasks in terms of cognitive demands.

Response 3:We sincerely appreciate the valuable comment. The following is our response to this comment.

In the introduction section, we have added the differences between 3D-MOT and traditional MOT, as well as the application advantages of 3D-MOT.

The revised part now reads: “Although the above-mentioned studies have extensively validated the tracking performance of athletes using the MOT task, most of them still employ the traditional two-dimensional planar MOT task. In the real environment, three-dimensional spatial information serves as a crucial clue for the visual system to judge and estimate the movement of objects. Individuals' attentional processing of dynamic information often combines multiple depth cues such as monocular and binocular depth cues for judgment and estimation, and these depth cues can provide individuals with a strong sense of depth perception[15]. ”(Lines 49-56, Page 5)

Reviewer#1: SPECIFIC COMMENTS

Comment 4:Introduction:Lines 60-75, Page 5: The introduction of the "Long-Term Athlete Development" (LTAD) model is relevant, but the connection between physical and cognitive development could be more explicitly linked to 3D-MOT performance.

Response 4:We sincerely appreciate the valuable comment. The following is our response to this comment.

In response to this comment, we have added relevant discussions in the introduction.

The revised part now reads:“Previous studies have suggested that the results of cognitive function tests can predict the success of top soccer players and that higher-order cognitive functions are believed to be related to talent identification and development in young soccer players[28]. During this period, it can thus be speculated that the development of dynamic visual attention might influence sport performance and their potential for future enhancement. In light of the importance of cognitive capabilities in sports[29], especially the function of dynamic visual attention in soccer, it is of paramount importance to comprehend the development of these processes in young players. ”(Lines 89-97, Page 7)

Reviewer#1: SPECIFIC COMMENTS

Comment 5: Methods: Lines 91-94, Page 6: The sample size for the 12-year-old group is small (n=16), which may limit the generalizability of the findings. The authors should address this limitation in the discussion.

Response 5:We sincerely appreciate the valuable comment. The following is our response to this comment.

In response to this comment, we have included a discussion in our study on the impact of the relatively small sample size in the 12-year-old group on the research results.

The revised part now reads�“Notably, no significant intergroup differences were observed between the age 12 group and other age groups. This might be attributed to limited statistical power resulting from the comparatively smaller sample size within the age 12 group (n = 16). Future studies could further expand the sample size, especially for the age group 12, to verify this hypothesis” ( Lines 339-343, Page 20)

Reviewer#1: SPECIFIC COMMENTS

Comment 6: Methods: Lines 108-122, Page 7: The description of the VR apparatus and stimuli is thorough, but the authors could provide more details on how the 3D-MOT task was validated for use with soccer players.

Response 6:We sincerely appreciate the valuable comment. The following is our response to this comment.

With regard to this comment, we add relevant research content and references to

Attachment Submitted filename: renamed_58796.docx

# Peer review: Decision Letter 1

**DOI:** 10.1371/journal.pone.0312051.r003

**Authors:** Rafael Oliveira

Dear Dr. Zhang,

Thank you for submitting your manuscript to PLOS ONE. After careful consideration, we feel that it has merit but does not fully meet PLOS ONE’s publication criteria as it currently stands. Therefore, we invite you to submit a revised version of the manuscript that addresses the points raised during the review process.

==============================

Dear authors, 

Thank you for addressing the comments of reviewers. Your manuscript improved its quality and the current form only needs minor revisions. 

Please see the comments by reviewers and the following:

-I would recommend writing in the third person (to avoid expressions like "we aimed", "we suggest" etc., etc.);

- abstract should present some statistics;

-please avoid using keywords already present in the title;

-please add a space when introducing citations in text (e.g., L28, the current form is "responses[1-4]", please amend to "responses [1-4]"). Please revise the full text;

-Introduction is too long. Please reduce the text to two and half pages at maximum;

-before introducing "Experiment 1", it is suggested describing Study Design where it should also be explained that 2 experiments were conducted;

-In data analysis (for both experiments), please describe the thresholds used to interpret the effect sizes. In addition, effect sizes should be presented for Bonferroni comparisons;

-Moreover, full reference of SPSS should be described. Please revise;

-L337, authors mentioned hypothesis but this was not explained in introduction. Thus, please clarify hypotheses of the study;

Best regards

==============================

Please submit your revised manuscript by Jun 16 2025 11:59PM. If you will need more time than this to complete your revisions, please reply to this message or contact the journal office at plosone@plos.org . When you're ready to submit your revision, log on to https://www.editorialmanager.com/pone/ and select the 'Submissions Needing Revision' folder to locate your manuscript file.

- A rebuttal letter that responds to each point raised by the academic editor and reviewer(s). You should upload this letter as a separate file labeled 'Response to Reviewers'.
- A marked-up copy of your manuscript that highlights changes made to the original version. You should upload this as a separate file labeled 'Revised Manuscript with Track Changes'.
- An unmarked version of your revised paper without tracked changes. You should upload this as a separate file labeled 'Manuscript'.

If applicable, we recommend that you deposit your laboratory protocols in protocols.io to enhance the reproducibility of your results. Protocols.io assigns your protocol its own identifier (DOI) so that it can be cited independently in the future. For instructions see: https://journals.plos.org/plosone/s/submission-guidelines#loc-laboratory-protocols . Additionally, PLOS ONE offers an option for publishing peer-reviewed Lab Protocol articles, which describe protocols hosted on protocols.io. Read more information on sharing protocols at https://plos.org/protocols?utm_medium=editorial-email&utm_source=authorletters&utm_campaign=protocols .

We look forward to receiving your revised manuscript.

Kind regards,

Rafael Oliveira

Academic Editor

PLOS ONE

**Journal Requirements:**

Please review your reference list to ensure that it is complete and correct. If you have cited papers that have been retracted, please include the rationale for doing so in the manuscript text, or remove these references and replace them with relevant current references. Any changes to the reference list should be mentioned in the rebuttal letter that accompanies your revised manuscript. If you need to cite a retracted article, indicate the article’s retracted status in the References list and also include a citation and full reference for the retraction notice.

**Additional Editor Comments:**

Dear authors,

Thank you for addressing the comments of reviewers. Your manuscript improved its quality and the current form only needs minor revisions.

Please see the comments by reviewers and the following:

-I would recommend writing in the third person (to avoid expressions like "we aimed", "we suggest" etc., etc.);

- abstract should present some statistics;

-please avoid using keywords already present in the title;

-please add a space when introducing citations in text (e.g., L28, the current form is "responses[1-4]", please amend to "responses [1-4]"). Please revise the full text;

-Introduction is too long. Please reduce the text to two and half pages at maximum;

-before introducing "Experiment 1", it is suggested describing Study Design where it should also be explained that 2 experiments were conducted;

-In data analysis (for both experiments), please describe the thresholds used to interpret the effect sizes. In addition, effect sizes should be presented for Bonferroni comparisons;

-Moreover, full reference of SPSS should be described. Please revise;

-L337, authors mentioned hypothesis but this was not explained in introduction. Thus, please clarify hypotheses of the study;

Best regards

[Note: HTML markup is below. Please do not edit.]

Reviewers' comments:

Reviewer's Responses to Questions

**Comments to the Author**

Reviewer #1: All comments have been addressed

**********

2. Is the manuscript technically sound, and do the data support the conclusions?

Reviewer #1: Yes

**********

3. Has the statistical analysis been performed appropriately and rigorously?

Reviewer #1: Yes

**********

4. Have the authors made all data underlying the findings in their manuscript fully available?

The PLOS Data policy

Reviewer #1: Yes

**********

5. Is the manuscript presented in an intelligible fashion and written in standard English?

Reviewer #1: Yes

**********

**Reviewer #1:**  General Comments

This revised manuscript investigates the developmental trajectory of three-dimensional multiple object tracking (3D-MOT) performance in young soccer players and examines age-related training effects. The authors have addressed several previous concerns, and the manuscript has improved in clarity and scientific rigor. The research provides valuable insights into perceptual-cognitive development in youth soccer players and offers potential applications for talent development and training programs.

Specific Comments

Abstract

1. Lines 19-20, Page 2: Need explicit acknowledgment in the abstract that data from players younger than 12 is missing, limiting conclusions about potential sensitive periods.

Introduction

2. Lines 32-36, Page 4: Requires more direct explanation of how 3D-MOT specifically relates to soccer performance compared to traditional MOT tasks.

3. Lines 60-67, Page 5: Needs better connection between previous 3D-MOT research findings and soccer-specific skills or performance measures.

4. Lines 89-92, Page 7: The claim about dynamic visual attention influencing sport performance requires additional supporting evidence.

Methods - Experiment 1

5. Lines 113-116, Page 8: The imbalanced sample sizes across age groups (especially n=16 for 12-year-olds) requires additional statistical justification.

6. Lines 142-143, Page 9: Citation needed to support the claim about technique effectiveness in measuring dynamic attention in soccer players.

7. Lines 159-165, Page 10: Need rationale for the specific parameters chosen in the adaptive procedure (30% increase, 20% decrease).

8. Lines 183-184, Page 11: Experiment dates should appear earlier in the methods as part of the study design description.

Results - Experiment 1

9. Lines 203-207, Page 12: Effect sizes should include standardized interpretations (small, medium, large) consistently throughout.

10. Lines 208-213, Page 13: Table 1 should include effect sizes and confidence intervals for all comparisons.

11. Lines 216-221, Page 14: Confidence intervals for the significant difference between 13 and 15-year-olds need consistency with other statistical reporting.

Methods - Experiment 2

12. Lines 236-248, Page 15: Need justification for the large effect size (.80) expected in the power analysis based on previous research.

13. Lines 252-255, Page 15: The short intervention period (15 sessions over 5 days) requires stronger justification.

Results - Experiment 2

14. Lines 274-276, Page 17: Figure 4 is referenced but not visible in the manuscript.

15. Lines 283-288, Page 17: Include interpretation of the effect size (η²=0.212) for the main effect of intervention on peak speed.

16. Lines 289-293, Page 17: Need interpretation of practical significance for average speed improvements.

Discussion

17. Lines 328-333, Page 19: The speculation about development peaks before age 12 lacks direct supporting data from this study and requires clearer acknowledgment of this limitation.

18. Lines 339-353, Page 20: Expand the discussion of peak speed sensitivity to include specific implications for training and assessment protocols.

19. Lines 354-376, Page 21: Neural mechanisms discussion needs more direct connection to the study's findings.

20. Lines 377-387, Page 22: More clearly identify the speculative nature of sensitive period discussions given the study design limitations.

Limitations and Future Directions

21. Lines 413-414, Page 24: Include specific recommendations for minimum sample sizes in future research based on power analyses.

Conclusion

22. Lines 416-426, Page 24: Address practical implications for coaches and training programs more explicitly.

References

Lines 428-592, Pages 25-33: The reference list would benefit from incorporating more recent and directly relevant studies on visual-perceptual training and cognitive development in young athletes. I recommend the authors consider including the following key references that would strengthen the theoretical framework and discussion:

1. Dhahbi, W., Chaabene, H., Pyne, D. B., & Chamari, K. (2024). Standardizing the quantification of external load across different training modalities: a critical need in sport-science research. International Journal of Sports Physiology and Performance, 19(11), 1173-1175. This would enhance your methodology section regarding training quantification.

2. Dhahbi, W., Briki, W., Heissel, A., Schega, L., Dergaa, I., Guelmami, N., et al. (2025). Physical Activity to Counter Age-Related Cognitive Decline: Benefits of Aerobic, Resistance, and Combined Training - A Narrative Review. Sports Medicine (Open). In press. This recent review provides valuable insights on cognitive development that would strengthen your discussion of age-related changes in perceptual-cognitive abilities.

3. Farhani, Z., Ghouili, H., Dhahbi, W., Ammar, A., Ben Aissa, M., Bouzouraa, M. M., ... & Ben Ezzeddine, L. (2025). Optimizing the number of players and training bout durations in soccer small‐sided games: Effects on mood balance and technical performance. European Journal of Sport Science, 25(3), e12208. This research on soccer-specific training would enhance your discussion of practical applications.

4. Dhahbi, W., Materne, O., & Chamari, K. (2025). Rethinking injury prevention strategies: joint-by-joint training approach paradigm versus traditional focused strengthening. Biology of Sport, 42(4), 59-65. This paradigm-shifting approach to training could inform your discussion of perceptual-cognitive training methodologies.

5. Ezzdine, L. B., Dhahbi, W., Dergaa, I., Ceylan, H. I., Guelmami, N., Ben Saad, H., ... & El Omri, A. (2025). Physical Activity and Neuroplasticity in Neurodegenerative Disorders: A Comprehensive Review of Exercise Interventions, Cognitive Training, and AI Applications. Frontiers in Neuroscience, 19, 1502417. This provides valuable insights on neuroplasticity mechanisms that would enhance your discussion of the neural basis for 3D-MOT performance improvements.

**********

what does this mean? ). If published, this will include your full peer review and any attached files.

If you choose “no”, your identity will remain anonymous but your review may still be made public.

**Do you want your identity to be public for this peer review?** For information about this choice, including consent withdrawal, please see our Privacy Policy

Reviewer #1: **Yes: ** Wissem Dhahbi

**********

[NOTE: If reviewer comments were submitted as an attachment file, they will be attached to this email and accessible via the submission site. Please log into your account, locate the manuscript record, and check for the action link "View Attachments". If this link does not appear, there are no attachment files.]

While revising your submission, please upload your figure files to the Preflight Analysis and Conversion Engine (PACE) digital diagnostic tool, https://pacev2.apexcovantage.com/ . PACE helps ensure that figures meet PLOS requirements. To use PACE, you must first register as a user. Registration is free. Then, login and navigate to the UPLOAD tab, where you will find detailed instructions on how to use the tool. If you encounter any issues or have any questions when using PACE, please email PLOS at figures@plos.org

# Author comment: Author response to Decision Letter 2

**DOI:** 10.1371/journal.pone.0312051.r004

28 May 2025

Dear Editors:

This is from one author of this submission of PONE-D-24-37901. Thank you very much for receiving your letter.

I have made the revisions in accordance with the comments from the editor and reviewers as mentioned in the email, and have re-uploaded the manuscript �the 'Revised Manuscript with Track Changes' and 'Response to Reviewers'.

Thank you very much, if you have any questions, please contact me in time.

Attachment Submitted filename: Response to Reviewers.docx

# Editor report: Decision Letter 2

**DOI:** 10.1371/journal.pone.0312051.r005

**Authors:** Rafael Oliveira

Three-dimensional multiple object tracking (3D-MOT) performance in young soccer players: age-related development and training effectiveness

PONE-D-24-37901R2

Dear Dr.  Yulu Zhang,

We’re pleased to inform you that your manuscript has been judged scientifically suitable for publication and will be formally accepted for publication once it meets all outstanding technical requirements.

Within one week, you’ll receive an e-mail detailing the required amendments. When these have been addressed, you’ll receive a formal acceptance letter and your manuscript will be scheduled for publication.

An invoice will be generated when your article is formally accepted. Please note, if your institution has a publishing partnership with PLOS and your article meets the relevant criteria, all or part of your publication costs will be covered. Please make sure your user information is up-to-date by logging into Editorial Manager at Editorial Manager®  and clicking the ‘Update My Information' link at the top of the page. If you have any questions relating to publication charges, please contact our Author Billing department directly at authorbilling@plos.org.

If your institution or institutions have a press office, please notify them about your upcoming paper to help maximize its impact. If they’ll be preparing press materials, please inform our press team as soon as possible -- no later than 48 hours after receiving the formal acceptance. Your manuscript will remain under strict press embargo until 2 pm Eastern Time on the date of publication. For more information, please contact onepress@plos.org.

Kind regards,

Rafael Oliveira

Academic Editor

PLOS ONE

Additional Editor Comments (optional):

Dear authors,

The authors improved their work based on comments made by reviewers and editor. Thus, the current form can be accepted for publication. During the final proofreading, authors should update SPSS reference accoridng to the most recent guidelines (which is still not the case). Morever, there are still some passages when "we" was written which should be amended.

Best regards

Reviewers' comments:

# Editor report: Acceptance letter

**DOI:** 10.1371/journal.pone.0312051.r006

**Authors:** Rafael Oliveira

PONE-D-24-37901R2

PLOS ONE

Dear Dr. Zhang,

I'm pleased to inform you that your manuscript has been deemed suitable for publication in PLOS ONE. Congratulations! Your manuscript is now being handed over to our production team.

At this stage, our production department will prepare your paper for publication. This includes ensuring the following:

* All references, tables, and figures are properly cited

* All relevant supporting information is included in the manuscript submission,

* There are no issues that prevent the paper from being properly typeset

You will receive further instructions from the production team, including instructions on how to review your proof when it is ready. Please keep in mind that we are working through a large volume of accepted articles, so please give us a few days to review your paper and let you know the next and final steps.

Lastly, if your institution or institutions have a press office, please let them know about your upcoming paper now to help maximize its impact. If they'll be preparing press materials, please inform our press team within the next 48 hours. Your manuscript will remain under strict press embargo until 2 pm Eastern Time on the date of publication. For more information, please contact onepress@plos.org.

If we can help with anything else, please email us at customercare@plos.org.

Thank you for submitting your work to PLOS ONE and supporting open access.

Kind regards,

PLOS ONE Editorial Office Staff

on behalf of

Prof Rafael Oliveira

Academic Editor

PLOS ONE
